# Influence of Graphite, Boron, Zirconium, and Hydroxyapatite Reinforcements on the Mechanostructure of Polyaryletheretherketone–Matrix Hybrid Composites

**DOI:** 10.3390/biomimetics11030203

**Published:** 2026-03-10

**Authors:** Bunyamin Aksakal, Cevher Kursat Macit, Yusuf Er, Merve Ayik

**Affiliations:** 1 Aircraft Airframe-Engine Maintenance, School of Aviation, Firat University, 23119 Elazig, Turkey; macitkursatcevher@gmail.com (C.K.M.); yusufer@firat.edu.tr (Y.E.); 2 Graduate School of Sciences, Firat University, 23119 Elazig, Turkey; mervehorlu@gmail.com

**Keywords:** polymer, hybrid composite, PEEK, graphite-boron-zirconium, mechanostructural properties

## Abstract

Polyether ether ketone (PEEK) is a high-performance thermoplastic with potential applications in aerospace, automotive, and biomedical components, owing to its exceptional specific strength, thermal stability, and biocompatibility. However, its moderate hardness and limited wear resistance in dry sliding severely constrain its use in highly loaded tribological contacts. In this study, PEEK-based reinforced hybrid composites were produced utilizing a powder metallurgy technique, with reinforcement fractions of 10 wt.% graphite (Gr), boron (B), hydroxyapatite (HAp), and zirconium (Zr). The processing sequence included homogeneous wet-mixing, uniaxial cold compaction at pressures of 10–30 MPa, and sintering at 250–300 °C. The composition and microstructures were characterized by X-ray diffraction (XRD), Fourier-transform infrared spectroscopy (FT-IR), scanning electron microscopy (SEM) and energy-dispersive X-ray spectroscopy (EDX). Mechanical and tribological performances were assessed by Vickers microhardness, uniaxial compression and dry sliding wear tests. The best-performing Gr-B hybrid composite increased hardness by 240% and compressive strength by 175% compared with unreinforced PEEK. Tribologically, boron-containing PEEK demonstrated up to a 34.7% reduction in the coefficient of friction and approximately a 90% drop in wear-induced mass loss compared with unreinforced PEEK. The resulting Gr-B-reinforced PEEK hybrids are excellent choices for demanding load-bearing and tribological components like aerospace bushings, automotive sliding elements, spinal cages, and orthopedic fixation devices in biomedical applications because of their balanced combination of high hardness, superior wear resistance, and high compressive strength.

## 1. Introduction

High-performance polymers are increasingly being adopted in tribology, structural mechanics, manufacturing, and even cost-driven engineering applications as lightweight alternatives to metals. Among them, polyether ether ketone (PEEK) is a high-temperature, semi-crystalline engineering thermoplastic whose application range is expanding rapidly. It is widely used as a metal substitute in demanding end-use components, particularly in the automotive, aerospace and industrial pump sectors [1,2,3]. In addition, its biocompatibility and radiolucency make it an attractive biomaterial for trauma, spinal and orthopedic implants. Compared with commodity engineering thermoplastics such as polyamide, e.g., PA66 and polyoxymethylene (POM), PEEK generally exhibits superior thermal stability and retains mechanical integrity at elevated service temperatures, while also offering comparatively lower wear under dry sliding in many metal–polymer tribosystems. In contrast to fluoropolymers such as PTFE, which can provide very low friction but are limited by comparatively low strength and load-carrying capacity, PEEK offers a more favorable balance between wear resistance, mechanical robustness, and high-temperature capability for structural tribological components, with reported continuous-use capability at 260 °C [4,5,6].

Nevertheless, its intrinsic wear resistance and mechanical performance under dry sliding contact are still insufficient for the most severe tribological environments, and further enhancement is required [7,8]. A widely adopted approach to improving the functional performance of PEEK is the development of composite and hybrid composite systems. The choice of reinforcement type, content and morphology is critical for tailoring mechanical, tribological and thermal properties to specific service conditions. In this context, the present study focuses on graphite (Gr), boron (B), zirconium (Zr) and hydroxyapatite (HAp) as reinforcing phases, selected based on their distinct physicochemical properties and compatibility with the PEEK matrix [9,10]. The combined use of ceramic, metallic and carbonaceous reinforcements is expected to enable a hybrid strengthening and self-lubricating mechanism, which is particularly attractive for high-load, dry sliding applications.

Graphite, the primary reinforcement in this work, is a layered carbon allotrope characterized by a lamellar, platelet-like structure composed of hexagonally arranged polycyclic carbon atoms. The weak van der Waals interactions between adjacent graphene layers confer excellent solid lubricant behavior, especially under high contact pressures and temperatures [11,12]. Accordingly, graphite-containing polymer composites have been widely reported to exhibit reduced coefficients of friction (COF) and improved wear resistance, thereby enhancing tribological performance in sliding contacts [13].

Boron, in elemental or compound form, is distinguished by its very high hardness, low density and excellent high-temperature stability. When incorporated into PEEK composites, boron-based reinforcements are expected to increase mechanical strength and wear resistance by acting as hard load-bearing phases and by reducing local plastic deformation and micro-cutting at the contact interface [14]. Additionally, boron-containing phases can modify the thermal stability and heat dissipation behavior of the composite, which is advantageous in applications involving exposure to elevated temperatures and friction-induced heating [15].

Zirconium (Zr) and its derivatives are widely employed in composite systems owing to their high corrosion resistance, good mechanical strength and chemical stability. The addition of Zr into PEEK-based composites can enhance structural integrity and wear resistance, particularly under aggressive service conditions frequently encountered in aerospace and biomedical environments [16]. Zirconium-based reinforcements have also been associated with improved fracture toughness and increased load-bearing capacity in polymer composites, mainly through crack deflection, crack bridging and constrained plastic deformation mechanisms [17].

Hydroxyapatite (HAp), a calcium phosphate-based bioceramic, has attracted considerable interest in biomedical engineering due to its excellent biocompatibility, bioactivity and osteoconductive behavior. Incorporating HAp into PEEK composites provides a route to combine the mechanical robustness and processability of PEEK with the biological affinity of HAp. Such hybrid systems are promising for orthopedic and dental implants, where simultaneous requirements of mechanical support, osseointegration and wear resistance must be satisfied. Furthermore, HAp particles can contribute to increased surface hardness and improved resistance to micro-abrasion, thereby supporting the long-term tribological performance of implantable devices [18].

From a processing standpoint, powder metallurgy (PM) offers a robust and cost-effective route for producing complex-shaped PEEK-based composite components with controlled dimensional tolerances and microstructures [11]. In particular, the use of different sintering techniques such as conventional furnace sintering, spark plasma sintering and microwave sintering plays a decisive role in determining densification behavior, crystallinity, interfacial bonding and, hence, the overall mechanical and tribological performance of the composites [17,18]. At elevated processing temperatures (i.e., the sintering/consolidation range of 250–300 °C employed in the present powder metallurgy route), chemical interactions between the polymer matrix and reinforcing phases may occur, potentially leading to new interfacial compounds or modified inter-phases that strongly influence load transfer and damage evolution [17,18].

The sintering of polymer grains can be described analogously to crack healing, where surface diffusion, viscous flow and chain interdiffusion govern neck growth and pore elimination. For semi-crystalline polymers such as PEEK, the final co-crystallization stage significantly reinforces interparticle interfaces through crystallization of adjacent polymer chains, thereby enhancing bulk stiffness and strength [14,15]. Control of sintering temperature, time and heating rate is therefore essential to optimize crystallinity, reduce residual porosity and promote strong matrix–reinforcement interfaces.

Numerous studies have examined the effect of different fillers and reinforcements on the properties of PEEK-based composites. Su et al. reported 35.8%, 25.4%, and 23.7% improvements in interlayer shear strength, flexural strength, and flexural modulus, respectively, by incorporating carbon nanotubes (CNTs) into carbon fiber (CF)/PEEK laminates [13]. Hernandez et al. investigated short CF-reinforced PEEK and showed that, although stiffness increased, the composites displayed reduced resistance to repeated impact loading [13]. Sumer et al. compared pure PEEK with 30 wt.% glass fiber (GF)/PEEK and found that the GF/PEEK composites exhibited a lower coefficient of friction (COF) and specific wear rate under both dry and water-lubricated conditions [15]. Pascual et al. demonstrated that the addition of single-walled CNTs (SWCNTs) and polyethersulfone (PES) to PEEK improved stiffness, strength and the glass transition temperature [15]. Ogasawara et al. reported that well-dispersed multi-walled CNTs (MWCNTs) in PEEK increased the elastic modulus by approximately 40% at 0.2% strain [17]. Song et al. showed that graphene oxide–silica (GO–Si) nanosheet-reinforced PEEK composites exhibit a low COF but comparatively high wear rates [14,15,16], while Puértolas et al. observed that graphene nanoplatelets decrease both the COF and the wear factor from 0.13 to 0.08 and from 22 × 10^−7^ to 4 × 10^−7^ mm^3^·N^−1^·m^−1^, respectively, but the ultimate tensile strength decreased from 105.6 to 87.5 MPa [16].

Despite the breadth of available literature on PEEK composites, there remains a notable gap concerning hybrid systems that simultaneously incorporate boron, hydroxyapatite and zirconium in combination with graphite within a PEEK matrix. In particular, systematic investigations of such multi-phase, ternary/quaternary hybrid composites produced by powder metallurgy, and their coupled microstructural, mechanical and tribological responses under dry sliding, are scarce.

The present study aims to address this gap by formulating and characterizing four different PEEK-based hybrid composite configurations containing Gr, B, HAp and Zr reinforcements. The composites are fabricated via cold pressing at different compaction pressures and sintering at two temperatures, followed by comprehensive microstructural and property evaluation. Microstructural features and phase compositions are examined using Fourier-transform infrared spectroscopy (FT-IR), X-ray diffraction (XRD), scanning electron microscopy (SEM) and energy-dispersive X-ray spectroscopy (EDX). Tribological behavior is assessed through hardness and dry sliding wear tests, while mechanical performance is quantified via uniaxial compression tests. By correlating processing parameters, microstructure and multi-scale properties, this work seeks to clarify the synergistic roles of Gr, B, HAp and Zr in enhancing the performance and extending the application window of PEEK-based hybrid composites.

## 2. Materials and Method

### 2.1. Materials and Composite Preparation

In this study, PEEK powder (99.9% Sigma-Aldrich, Darmstad, Germany) was used as the matrix material, while graphite (Gr), boron (B), hydroxyapatite (HAp) and zirconium (Zr) powders (each 99.9% nominal purity (Nanografi, Ankara, Turkey) served as reinforcements. Table 1 shows the composite groups and the sample groups. The average particle size of the PEEK matrix was <50 µm, and the reinforcing powders exhibited particle sizes of <35 µm (Table 2). The particle size mismatch between matrix and reinforcement is consistent with previous powder metallurgy studies, where fine secondary particles occupy interstitial sites between coarser matrix grains, thereby enhancing packing density, mechanical interlocking and stress transfer across the interface [13,14].

To ensure full traceability and reproducibility, the purity/grade, particle size, and manufacturer (city/country) information for the matrix and reinforcement powders used to prepare the compositions are listed in Table 2.

To promote a homogeneous distribution of the reinforcements within the PEEK matrix, a wet-mixing route was adopted. The required amounts of PEEK and reinforcement powders, according to the compositions given in Table 1, were first weighed and premixed. Absolute ethanol (ethyl alcohol, ≥99.9% (CAS 64-17-5, ISOLAB, Germany)) was then added as a liquid carrier and dispersing medium to reduce van der Waals-driven agglomeration of the fine particles and to improve wetting of the reinforcement surfaces by the polymer powder. Citric acid (≥99.5%, CAS 77-92-9; Sigma-Aldrich, Darmstadt, Germany) was introduced as a chelating and binding agent at a concentration of 1.0 wt.% with respect to the total powder mass (PEEK + reinforcements); it modifies the surface chemistry of ceramic and metallic particles, improves their compatibility with the organic matrix, and promotes stronger interfacial adhesion by forming coordination or hydrogen bonds at the powder–powder interface.

The resulting slurry was subjected to mechanical mixing in a magnetic grinder (Restch-PM100, Germany) at 300 rpm for 3 h. This intensive mixing step ensured de-agglomeration of the fine reinforcements, disruption of soft clusters and intimate contact between PEEK and Gr/B/HAp/Zr particles. In particular, extended grinding was employed to compensate for the relatively large PEEK particle size and to minimize the risk of incomplete or non-uniform bonding at the matrix–reinforcement interface. After mixing, the slurry was dried in a vacuum oven (Protherm, Germany) at 90 °C for 12 h. Vacuum drying served to remove ethyl alcohol completely, suppress oxidation and moisture uptake, and stabilize the powder blend prior to compaction.

The dried composite powders were gently crushed and sieved, then loaded into a cylindrical steel die lubricated with zinc stearate ((CH_3_(CH_2_)_16_COO)_2_Zn; CAS 557-05-1; Sigma-Aldrich, Germany) to reduce die wall friction and prevent powder–die adhesion. Uniaxial cold compaction was carried out in a hydraulic press (SPECAC 25t-Orpington, UK) at two different pressures, 10 MPa and 30 MPa, to investigate the effect of green density on subsequent densification and properties.

These pressure levels were selected to represent two distinct compaction regimes, enabling a controlled assessment of how green density and interparticle contact area govern solid-state bonding during sintering: 10 MPa provides a baseline level of particle rearrangement and green strength, whereas 30 MPa yields a markedly higher packing density and contact fraction, which are critical for neck formation and pore closure in powder-processed polymers. Cold pressing was performed at room temperature, i.e., well below the sintering temperatures, and primarily aimed at achieving sufficient green strength, eliminating large pores and establishing close particle contact prior to thermal bonding.

Following compaction, the green specimens were sintered in an argon atmosphere to avoid oxidative degradation of PEEK and the reinforcements. Sintering was conducted at two temperatures, 250 °C and 300 °C, with a heating rate of 5 °C min^−1^, a holding time of 90 min at the peak temperature, and subsequent furnace cooling. The two sintering temperatures were chosen to probe the temperature sensitivity of PEEK solid-state sintering while remaining outside a melt-processing regime: both values lie well above the glass transition temperature of PEEK, enabling significant chain mobility and interdiffusion at particle contacts, yet remain sufficiently below the melting point (343 °C) to avoid melt flow, dimensional distortion and potential reinforcement segregation. Specifically, 250 °C serves as a lower-bound condition where diffusion-driven bonding is feasible but kinetically limited, whereas 300 °C provides enhanced segmental mobility and accelerated neck growth for a comparative evaluation of densification and interfacial development under otherwise identical thermal schedules. These temperatures were selected to lie above the glass transition temperature of PEEK but below its melting point (343 °C), enabling solid-state sintering via chain mobility, surface diffusion and interparticle neck growth without excessive melt flow or shape distortion. Under these conditions, co-crystallization of adjacent polymer chains across particle boundaries contributes to strengthening of interparticle necks and improvement in bulk mechanical performance [14,15].

The total reinforcement content was fixed at 10 wt.% in all composite formulations, with the remaining 90 wt.% being PEEK. This constant level was chosen as an optimum compromise between mechanical/tribological enhancement and microstructural/process stability. Research on PEEK- and polymer-based hybrid composites shows that reinforcement levels below ~5 wt.% result in modest gains in stiffness, hardness, and wear resistance due to insufficient load-bearing contribution. Higher concentrations (15–20 wt.%) can cause particle agglomeration, poor interfacial bonding, and stress concentration sites, resulting in decreased toughness, compressive strength, fatigue resistance, and increased brittleness [19,20]. Thus, by restricting the total reinforcement fraction to 10 wt.%, a sufficiently high surface area-to-volume ratio of hard and/or lubricating phases is achieved to improve hardness, strength and wear resistance, while maintaining a continuous polymer matrix and avoiding severe clustering. This level also helps preserve adequate processability in the powder metallurgy route, allowing good particle packing, efficient compaction and defect-minimized sintering. Previous hybrid composite studies have reported that 10 wt.% reinforcement provides an effective balance between mechanical performance, dispersion homogeneity and structural integrity, particularly in systems where multiple phases must be co-dispersed within an organic matrix.

**Figure 1:**
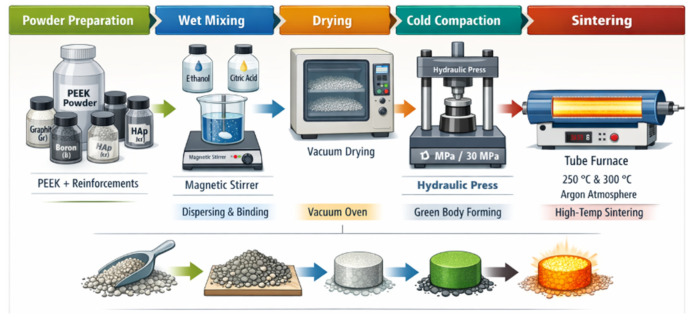
Experimental flow chart.

For each composition (Ref, A, B, C and D), four processing conditions (10 MPa—250 °C, 10 MPa—300 °C, 30 MPa—250 °C, 30 MPa—300 °C) were investigated, yielding a total of 20 samples. A schematic representation of the overall powder preparation, mixing, compaction and sintering sequence is illustrated in Figure 1. The detailed nomenclature and processing parameters of the produced sample groups are given in Table 3.

**Figure 2:**
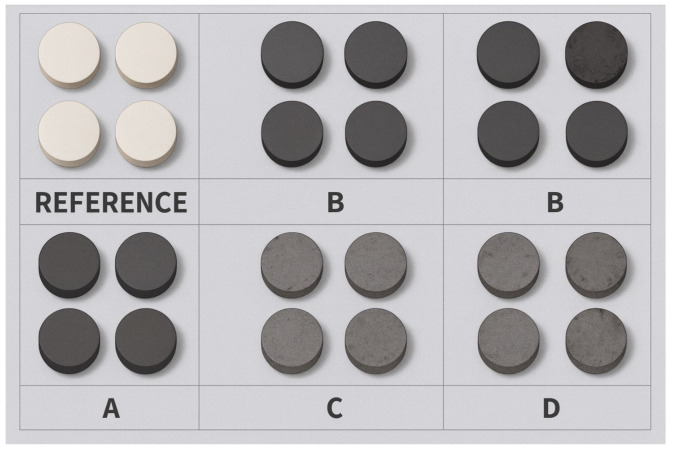
Reference (PEEK) and reinforced PEEK samples produced by cold pressing and sintering.

The samples obtained after cold pressing were then sintered in a tube furnace (Thermomac CMF7, Istanbul, Turkey) at two different temperatures (250 and 300 °C) for 4 h. XRD, FT-IR, SEM and EDS analyzes were performed to examine the microstructures of the produced samples. The tribological properties of the hybrid composites that were ready for testing were examined using wear and hardness tests, while mechanical properties were evaluated using compression tests. Test specimens prepared by two different pressing and sintering processes are shown in Figure 2.

### 2.2. Microstructure, Hardness, Tribology and Characterization

The characterization methodology for the manufactured hybrid PEEK composites was created to determine the effect of reinforcement type, compaction pressure, and sintering temperature on microstructural integrity, crystallographic behavior, and functional performance. All analyses were carried out on specimens that had been pressed, sintered, ground, and polished to achieve uniform surface quality.

### 2.3. Microstructural Analysis

The microstructure of the composites was examined using SEM (Sigma 300, Zeiss, Germany), which enabled detailed observation of particle morphology, dispersion uniformity and interfacial bonding between PEEK and the reinforcement phases. The distribution of Gr, B, HAp and Zr particles within the polymer matrix was assessed to identify possible agglomeration, porosity, or particle pull-out induced by processing conditions. Energy-dispersive X-ray spectroscopy (EDS) was utilized concurrently to ascertain the elemental composition and spatial distribution of the reinforcement phases, offering insights into chemical uniformity and reinforcement-matrix compatibility throughout the microstructure.

### 2.4. Structural and Phase Characterization

X-ray diffraction (XRD - Empyrean, Malvern Panalytical, The Netherlands; CuKα radiation, λ = 1.5406 Å) was used to identify the crystalline phases present in the pure and reinforced-PEEK samples. Diffraction patterns were collected within the 2θ range of 10–80°, enabling the detection of characteristic PEEK reflections together with diffraction peaks associated with graphite, boron, hydroxyapatite and zirconium. Variations in peak intensity and breadth were evaluated to determine the influence of sintering on crystallinity and lattice order. Fourier-transform infrared spectroscopy, FT-IR (Thermo Sci Nicolet iS5, MA, USA), was used to analyze vibrational modes in the range of 4000–500 cm^−1^, capturing the spectral signatures of the polymer backbone and identifying changes attributable to interactions with the reinforcing phases. FT-IR measurements confirmed the presence of characteristic functional groups and provided evidence of interfacial chemical compatibility.

### 2.5. Hardness Tests

Hardness measurements were carried out in accordance with ASTM E92-17 [21] using a dynamic hardness tester (Onalkon 200HRS, Bursa, Turkey) [22]. To eliminate the influence of substrate effects and ensure that the indentation remained fully within the composite layer, the penetration depth was restricted to less than one-tenth of the total specimen thickness. A load of 100 g with a 5 s dwell time was applied, and five independent measurements were obtained from each sample in order to establish representative hardness values. These measurements reflect the combined influence of reinforcement dispersion, local densification and matrix–filler interfacial strength on resistance to localized plastic deformation.

### 2.6. Tribological Characterization

Dry sliding wear tests were performed using a reciprocating tribometer (Elkimak, Elazığ, Turkey) at a sliding velocity of 1 ms^−1^, following ASTM G113-05 [21]. All samples were cleaned with acetone before and after testing to ensure consistent surface conditions. Each experiment was conducted on a fresh counterface to eliminate prior transfer layer effects. Weight loss was measured with an analytical balance of 10^−4^ g precision, enabling accurate determination of wear-induced mass changes. The coefficient of friction was calculated from the steady-state region of the friction–distance curve. After testing, the worn surfaces were examined using an optical digital microscope (Olympus CX23, Tokyo, Japan), (10 × 220 × magnification) to identify wear-track morphology, debris characteristics and dominant wear mechanisms. Additional SEM analysis was performed on gold-sputtered worn surfaces to observe micro-grooving, fatigue features, micro-cracking and the presence of transfer films, thereby providing a detailed understanding of how reinforcement phases influence the tribological response.

### 2.7. Compression Testing

Compressive mechanical behavior was evaluated using a universal testing machine (BESMAK, 50 kN, Ankara, Turkey) in accordance with the ASTM D3410/D3410M-03 standard [23]. Cylindrical specimens with dimensions of 13 mm in height and 9 mm in diameter were tested at a constant crosshead speed of 0.5 mm/min. The resulting stress–strain curves provided quantitative assessments of elastic modulus, compressive yield behavior and maximum compressive strength. These parameters offer direct insight into the contribution of reinforcement phases to load transfer, matrix stiffening, and damage evolution mechanisms during axial compression.

## 3. Results and Discussion

### 3.1. Phase Identification and Crystallographic Analysis (XRD)

**Figure 3:**
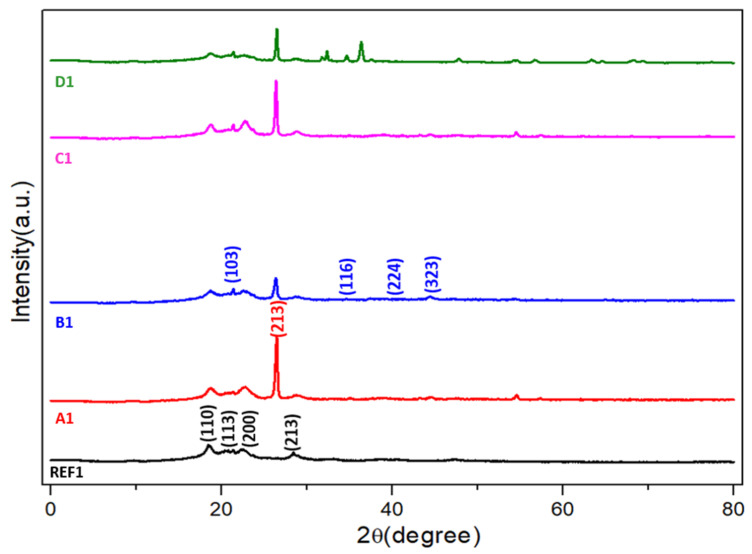
XRD analysis of the reference and reinforced PEEK samples.

XRD analysis was performed to determine the crystalline characteristics, phase composition and structural evolution of the reinforced hybrid PEEK composites. The diffraction patterns were obtained from fully sintered and metallographically prepared specimens, without Bakelite embedding, to ensure accurate phase identification and to prevent extraneous background signals. Figure 3 presents the XRD spectra of all composite formulations, where characteristic reflections of the PEEK matrix and the incorporated reinforcement phases can be clearly distinguished. The presence of the expected C, B, HAp and Zr diffraction peaks confirms the successful incorporation of each reinforcing phase within the polymer matrix, consistent with previous reports on polymer–ceramic and polymer–carbon hybrids [24,25,26,27]. All diffraction plots and other graphical representations were generated using OriginPro(10.05) software (OriginLab Corp, MA, USA).

PEEK is known to exhibit a semi-crystalline structure with an orthorhombic crystalline phase [28]. In all samples, prominent reflections at approximately 2θ ≈ 20° associated with the (110), (113) and (200) crystallographic planes were observed, indicating the retention of the characteristic ordered structure of PEEK. A distinct peak around 2θ ≈ 29°, corresponding to the (213) plane, was also present, in agreement with earlier studies [29]. These reflections confirm that the reinforcement procedure did not disrupt the intrinsic crystalline framework of the polymer. However, subtle variations in peak intensity and broadening among the reinforced samples suggest that the degree of crystallinity may have been locally modified by the presence of the fillers through nucleation, restriction of polymer chain mobility or microstrain generation.

The graphite-containing composites exhibited a sharp and intense peak at approximately 2θ ≈ 26°, corresponding to the (002) basal plane of graphite, in accordance with JCPDS 41-1487 [30,31]. This peak indicates the layered graphitic structure and demonstrates that the graphite particles retained their crystalline identity after processing. The narrowness and defined shape of this peak may also suggest partial alignment or preservation of coherent graphitic domains, implying that graphite lamellae were well-dispersed and structurally integrated within the PEEK matrix.

Elemental boron reinforcement was confirmed by the appearance of characteristic peaks near 2θ ≈ 27°, 38°, 48° and 55°, representing the (103), (116), (224) and (323) crystallographic planes. These peaks appeared with relatively low intensities. Such weak reflections are likely attributable to the partially amorphous nature of boron, its inherently low scattering factor or limited long-range structural order within the polymer matrix [32,33]. Similar behavior has been reported in prior work on boron-containing polymer composites, where boron tended to form nano-scale domains with short-range structural order rather than large crystalline aggregates. Hydroxyapatite and zirconium phases were also successfully identified through their respective characteristic diffraction peaks, though they appeared with modest intensity [34,35,36,37]. The reduced peak intensity suggests fine-scale dispersion of HAp and Zr within the polymer matrix and the absence of large agglomerates. This fine dispersion is advantageous for interfacial bonding, since nano-scale reinforcements typically provide more effective stress transfer and stronger interfacial interactions with the polymeric chains.

Overall, the XRD patterns demonstrate that the hybridization strategy preserved the essential PEEK crystalline structure while enabling the incorporation of multiple reinforcing phases without phase degradation or undesirable chemical reactions. The consistent appearance of reinforcement-related peaks at the expected 2θ positions indicates that the powders remained chemically stable throughout the powder metallurgy process. Minor peak shifts and local broadening observed in some samples may be associated with residual microstrain, interfacial bonding effects, polymer chain confinement or localized structural distortions induced during sintering [38,39,40].

These findings are consistent with prior studies on hybrid PEEK systems, where the simultaneous presence of carbonaceous and ceramic reinforcements enhanced mechanical and tribological properties while maintaining the fundamental structural integrity of the matrix [29,41,42]. The combined crystalline signatures observed here support the conclusion that the reinforcements were successfully and uniformly integrated into the PEEK matrix, establishing the structural foundation for the mechanical and tribological improvements reported in the subsequent sections.

### 3.2. Functional Group and Bonding Analysis (FT-IR)

**Figure 4:**
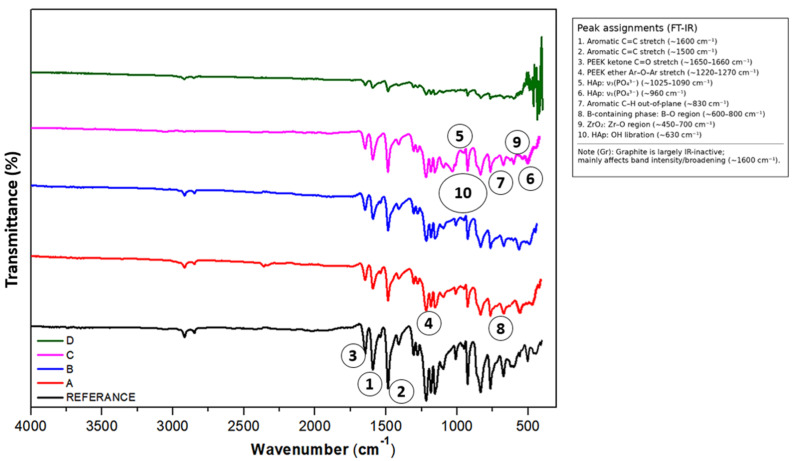
FT-IR spectrum of the samples pressed at 30 MPa and sintered at 300 degrees.

FT-IR spectroscopy was employed to elucidate the chemical structure, molecular bonding characteristics and potential interfacial interactions present in the synthesized hybrid PEEK composites. Figure 4 presents the representative FT-IR spectra, where the characteristic vibrational modes of the PEEK backbone and the additional spectral contributions of Gr, B, HAp and Zr reinforcements were identified and evaluated in light of prior literature.

The spectrum of neat PEEK shows the well-established absorption bands associated with its semi-aromatic polymeric structure. Strong peaks at approximately 1500 cm^−1^ and 1600 cm^−1^ correspond to aromatic ring stretching vibrations, confirming the integrity of the benzene-based backbone of PEEK [43]. Additional bands between 900 and 670 cm^−1^ arise from C–H out-of-plane bending modes typical of substituted aromatic systems [44], reflecting the inherent rigidity and thermal stability that characterize high-performance PEEK formulations. Absorption bands located in the 1225–950 cm^−1^ region are attributed to C–H in-plane bending modes coupled with aromatic skeletal vibrations. The structural features most critical to PEEK’s outstanding chemical resistance, namely the ether (–O–) and ketone (C=O) linkages, manifest as distinct peaks near 1250 cm^−1^ and 1730 cm^−1^, respectively. These functional groups form the fundamental segmental motifs responsible for the polymer’s resistance to thermal degradation and its ability to maintain mechanical integrity under demanding service conditions. Weak aliphatic C–H stretching vibrations detected between 2800 and 3000 cm^−1^ suggest minor variations in chain mobility induced by reinforcement incorporation, potentially linked to localized chain scission or physical confinement at filler interfaces [44].

In graphite-reinforced composites, subtle yet meaningful spectral modifications were observed. The enhancement of vibrational features in the C=C stretching region (~1600 cm^−1^) indicates π–π interactions between graphitic layers and the aromatic rings of PEEK [45]. These non-covalent interactions may promote improved load transfer, stiffening of the local polymer environment and alterations in electronic structure. Increased intensity in the C–H bending region between 670 and 900 cm^−1^ is consistent with prior observations for carbonaceous fillers in polymer matrices and reflects the presence of graphitic domains embedded within the PEEK network [46]. The incorporation of boron introduces new vibrational modes in the 600–800 cm^−1^ range. These features correspond to B–O stretching vibrations and are characteristic of boron-oxide or boron-containing ceramic phases [47]. Their presence confirms that boron has been successfully integrated into the composite structure and may exist partly in oxidized form. Weak absorptions in the 1200–1300 cm^−1^ region suggest possible B–C and B–N bonding environments, as previously described for boron-modified polymer systems [48]. Such bonding configurations can influence local stiffness, thermal stability and resistance to deformation, supporting the mechanical improvements documented in subsequent sections.

Hydroxyapatite-containing composites exhibit the distinctive vibrational signatures of phosphate groups, including the ν_1_ PO_4_^3−^ symmetrical stretching at ~960 cm^−1^ and the ν_3_ PO_4_^3−^ asymmetric stretching modes between 1025 and 1090 cm^−1^ [49,50,51]. The broad O–H stretching band in the 3200–3600 cm^−1^ region further confirms the presence of HAp [51] and demonstrates that the bioceramic maintains its structural identity within the polymer matrix. The absence of significant peak shifts suggests that HAp does not undergo chemical decomposition during sintering, which is important for biomedical applicability, particularly in load-bearing or osseointegrative implant components.

The zirconium-reinforced composites display additional absorption features in the 450–700 cm^−1^ region that can be assigned to Zr–O stretching modes [52]. These bands are consistent with vibrational signatures reported for ZrO_2_-reinforced polymer systems [53]. The presence of Zr-based vibrational modes, together with mild broadening of the C=O stretching band near 1730 cm^−1^, suggests weak coordination interactions or hydrogen-bonding tendencies between Zr^4+^ centers and oxygen-containing functional groups in PEEK. Such interactions may enhance interfacial adhesion and improve the load-bearing efficiency of the composite.

The FT-IR results collectively demonstrate that the fundamental chemical structure of PEEK remains intact after hybrid reinforcement and powder-metallurgical processing. Characteristic PEEK bands are preserved, indicating chemical stability of the polymer backbone. Simultaneously, the presence of distinct absorption bands attributable to Gr, B, HAp and Zr confirms the successful incorporation of all reinforcement phases without undesirable chemical reactions or degradation. Subtle changes in peak intensity, broadening and position suggest the presence of interfacial interactions, whether physical, electrostatic or coordinative, between the polymer chains and the reinforcement phases. These molecular-level interactions are likely to contribute to variations in crystallinity, stiffness, energy dissipation mechanisms and tribological behavior, as corroborated by the mechanical and wear results. This spectroscopic evidence is entirely consistent with earlier studies demonstrating that ceramic and carbonaceous reinforcements can enhance the physicochemical performance of PEEK-based composite systems [40,54,55].

In summary, FT-IR spectroscopy verifies that the hybrid reinforcement approach maintains the structural integrity of the PEEK matrix while enabling the integration of functional secondary phases. The spectral modifications identified provide a foundation for establishing clear structure–property correlations, particularly in the context of applications requiring superior strength, wear resistance and biocompatibility.

### 3.3. Microstructural and Elemental Characterization (SEM/EDX)

**Figure 5:**
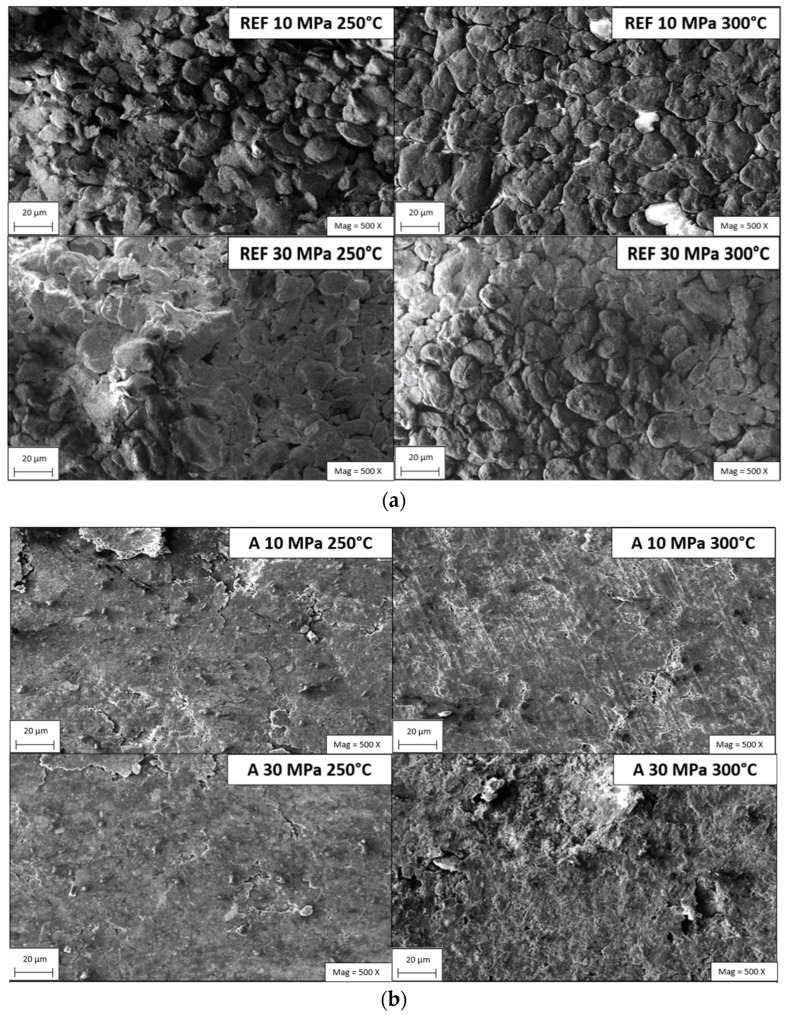

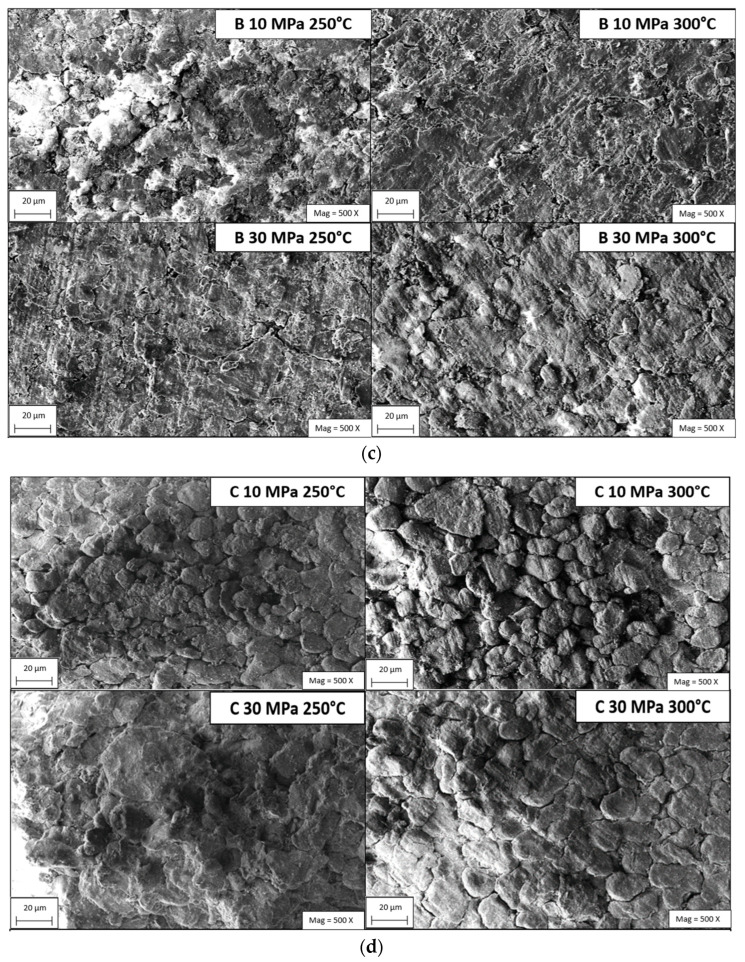

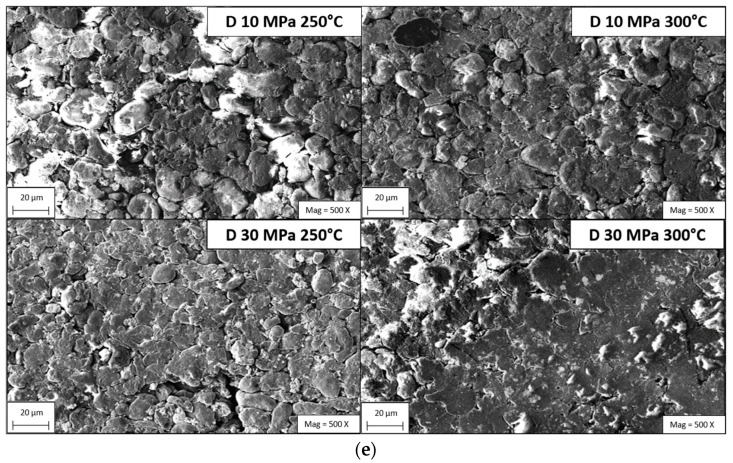
SEM micrographs of the produced PEEK-based hybrid composites: (**a**) Ref, (**b**) A, (**c**) B, (**d**) C, and (**e**) D groups.

**Figure 6:**
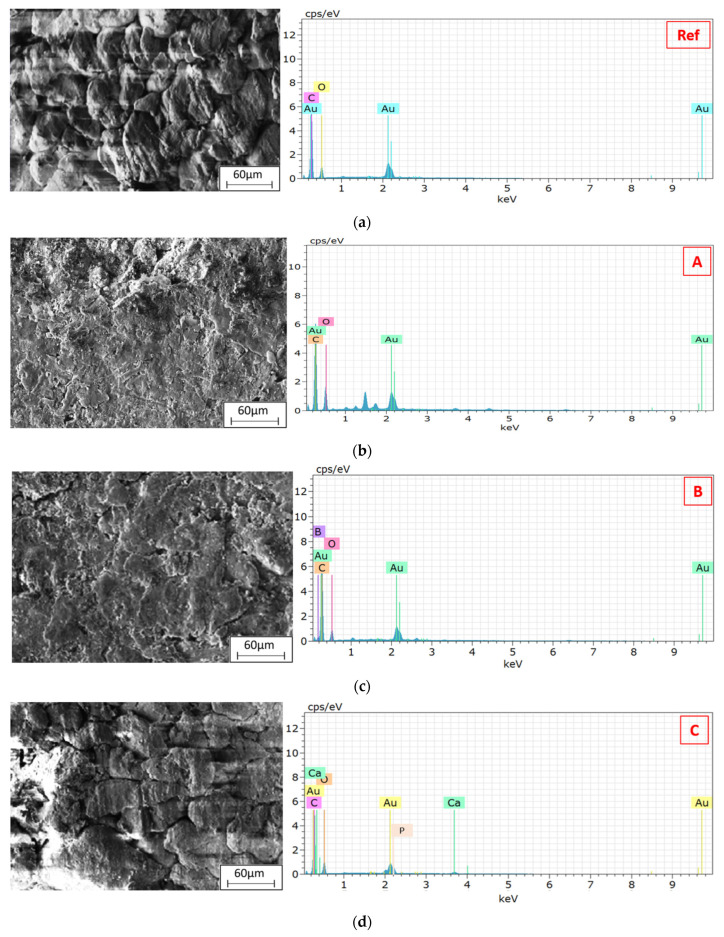

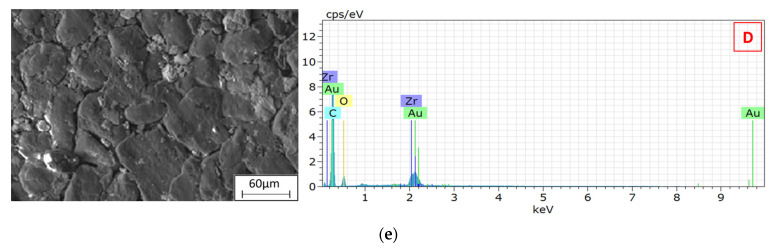
EDX spectra of produced hybrid composites (**a**) Ref, (**b**) A, (**c**) B, (**d**) C, (**e**) D groups.

**Figure 7:**
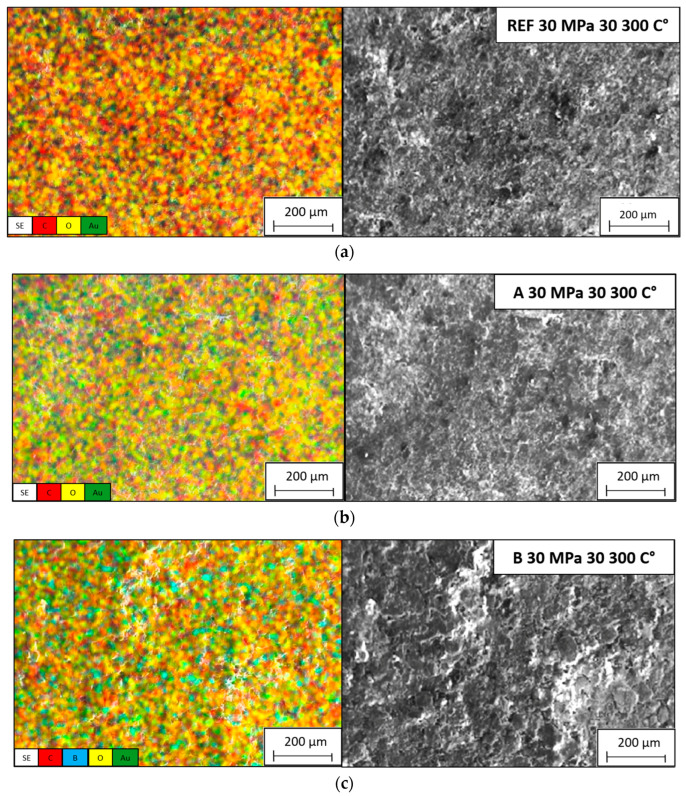

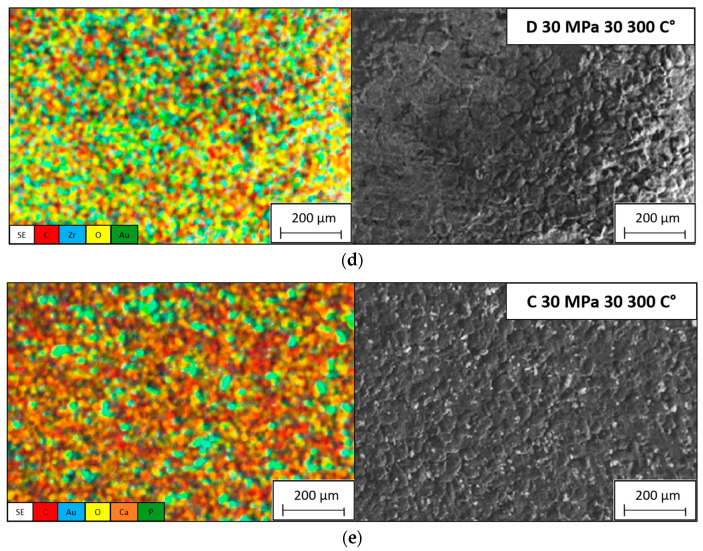
EDX maps of produced hybrid composite groups: (**a**) Ref, (**b**) A, (**c**) B, (**d**) C, and (**e**) D.

SEM and EDX analyses were employed to investigate the microstructural morphology, dispersion uniformity and elemental distribution of the hybrid reinforcements within the PEEK matrix. Representative SEM micrographs, obtained after metallographic polishing and subsequent gold sputter coating, are presented in Figure 5, Figure 6 and Figure 7 together with the corresponding EDX elemental maps. These combined observations provide critical insight into the spatial arrangement of the reinforcements, the integrity of the matrix–filler interfaces and the overall efficiency of phase integration.

The SEM micrographs in Figure 5 provide the first-level evidence for the effectiveness of the applied powder metallurgy route in establishing a compact and coherent hybrid microstructure. Across all compositions, the reinforcement phases appear embedded within the PEEK matrix without pronounced macro-scale segregation, indicating that wet-mixing, vacuum drying and subsequent solid-state consolidation achieved an adequate dispersion state [6,8,56,57,58]. Importantly, the micrographs also allow a qualitative assessment of interfacial integrity: regions exhibiting intimate particle–matrix contact and limited interfacial gaps are consistent with effective mechanical interlocking and improved load transfer, whereas local voids or weakly bonded particle boundaries would be expected to act as stress concentrators and potential pull-out sites during mechanical loading and sliding. In this context, the SEM observations support the microstructure–property correlations discussed in the subsequent sections, particularly for hardness and wear behavior, where porosity level and interface continuity play a decisive role [6,8,56,57,58]. To confirm that the observed microstructural features correspond to the intended reinforcement chemistries (Gr, B, HAp and Zr) and to validate compositional consistency among the groups, the elemental constitution was next examined using EDX point/spectrum analysis (Figure 6).

The EDX spectra presented in Figure 6 verify the presence of the targeted reinforcement-related elements in the corresponding composite groups and provide compositional confirmation complementary to SEM morphology. In particular, the dominant carbon signal is expected due to the polymer matrix and graphite-containing formulations, while the additional characteristic signals associated with boron-containing, Ca–P–O-rich (HAp) and Zr-containing phases confirm that the hybrid compositions were retained after mixing, compaction and sintering without evidence of gross contamination or phase loss [6,8,56,57,58]. However, EDX spectra alone primarily reflect local/spot compositional information and do not fully capture spatial homogeneity. Therefore, to evaluate whether these elements are uniformly distributed across the examined microstructural fields, an essential criterion for minimizing local stress concentrations, suppressing reinforcement clustering, and stabilizing tribological performance, the elemental distribution was further assessed using EDX mapping, as shown in Figure 7.

The SEM micrographs reveal that the reinforcing phases Gr, B, HAp and Zr are uniformly dispersed within the PEEK matrix, indicating that the adopted processing route comprising controlled mixing, cold compaction and sintering was effective in suppressing large-scale phase segregation and extensive agglomeration. Achieving such homogeneous filler distribution is typically challenging in polymer matrix composites, since the high surface energy of micro- and nano-scale reinforcements tends to promote clustering, which can severely deteriorate mechanical and tribological performance [16,59]. In the present system, the deliberate particle size disparity between the matrix (average particle size ~44 µm) and the reinforcements (typically < 10 µm) facilitated efficient packing; fine particles preferentially occupy interstitial voids between coarser PEEK grains, thereby increasing the effective contact area, improving interfacial mechanical interlocking and enhancing load transfer at the matrix–reinforcement interface [45,46]. This microstructural interpenetration underpins the formation of a denser and structurally coherent composite architecture.

A pronounced microstructural evolution with sintering temperature was also observed. At higher sintering temperatures, the pore size decreases and the pore morphology transitions from irregular, interconnected cavities to more isolated and spheroidal pores. This behavior is consistent with previous observations in polymer–ceramic hybrid systems [47] and is attributed to the increased chain mobility of PEEK and accelerated sintering kinetics, which promote viscous flow, neck growth and interfacial diffusion between particles. The reduction in interconnected porosity is particularly beneficial, as it limits pathways for moisture ingress and mechanical weakness, thereby improving structural homogeneity and long-term reliability under service conditions.

EDX analysis was conducted to verify the elemental composition and spatial distribution of the reinforcement phases. The recorded spectra and elemental maps exhibit distinct and well-dispersed signals corresponding to C, B, P, Ca and Zr, confirming the successful incorporation of Gr, B, HAp and Zr into the PEEK matrix without pronounced phase segregation [48,49]. The carbon signal, associated predominantly with graphite, confirms the presence and broad distribution of the lubricating phase. Graphite is known to reduce friction and wear through its inherent lamellar structure, which can form a protective tribofilm under sliding conditions [50,51]. The layered graphitic morphology additionally acts as a barrier to crack propagation and can improve fatigue resistance by redistributing local stresses. The consistent detection of boron in the EDX maps corroborates its homogeneous dispersion throughout the matrix. Due to its high hardness, chemical inertness and low density, boron contributes to strengthening and hardening of the composite and enhances its wear resistance by impeding localized plastic deformation and micro-cutting processes [40]. Phosphorus and calcium signals confirm the presence of hydroxyapatite. The refined and well-distributed HAp phase is particularly important for biomedical applications, as it is associated with osteoconductivity and biocompatibility, thereby supporting bone integration while helping maintain mechanical integrity [40,53]. The uniform distribution of HAp suggests favorable conditions for a consistent biological interface and uniform bone–implant interactions.

The detection of zirconium further validates its successful integration within the PEEK matrix. Zr is widely recognized for improving thermal stability, hardness and mechanical robustness in high-performance composites [54,55]. In addition, Zr-containing particles can act as nucleation sites during the crystallization of PEEK, which may promote more refined crystalline morphologies and enhance dimensional stability. The relatively uniform Zr distribution observed in the EDX maps indicates good chemical compatibility and strong interfacial bonding between Zr-rich regions and the surrounding polymer, both of which are essential for maintaining mechanical performance under cyclic or impact loading.

Taken together, the SEM and EDX results confirm that the adopted hybrid reinforcement strategy produces a microstructure characterized by well-dispersed reinforcement phases, minimal agglomeration and enhanced interfacial coherence. The observed reduction in porosity, the refined dispersion of reinforcements and the strong matrix–filler interfaces can be directly linked to the improved mechanical, thermal and tribological properties reported for these composites. Such morphological uniformity is crucial for mitigating localized stress concentrations that could otherwise trigger premature failure, and it underlies the observed improvements in stiffness, compressive strength and wear resistance.

These findings are in good agreement with previous studies on PEEK-based hybrid composites, which have shown that optimal dispersion and interfacial compatibility between reinforcements and polymer matrix are key to achieving superior mechanical durability and functional reliability [28,29,30,31]. The SEM and EDX analyses presented here provide compelling microstructural evidence of the successful co-integration of Gr, B, HAp and Zr within the PEEK matrix and highlight the synergistic role of these phases in enhancing the overall performance of the hybrid composites. This level of structural and compositional uniformity is a prerequisite for high-performance applications such as biomedical implants, aerospace components and advanced engineering systems that demand long-term dimensional stability, mechanical resilience and tribological endurance.

### 3.4. Hardness

Hardness is a principal mechanical parameter that reflects a material’s resistance to localized plastic deformation, abrasion, cutting and surface damage. In polymer-based composite systems, hardness serves as a direct indicator of surface strengthening and is widely correlated with tribological performance under conditions involving repeated sliding, asperity interaction and high contact stresses [48,49]. Although increases in hardness are often associated with improved wear resistance, the relationship between the two is influenced by a combination of microstructural and mechanical factors, including fracture toughness, matrix–filler interfacial adhesion, polymer thermal transitions, filler geometry and load distribution mechanisms [51,52,53].

**Figure 8:**
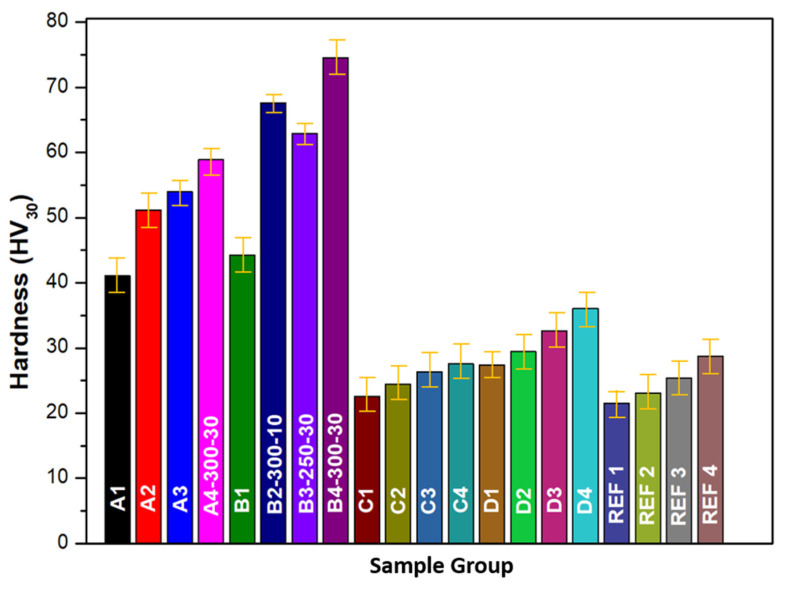
Hardness values of the produced hybrid composites.

Figure 8 presents the Vickers microhardness values measured at five distinct points on each specimen to ensure statistical reliability. All reinforced composites exhibited markedly higher hardness values compared to unreinforced PEEK, demonstrating the effectiveness of the hybrid reinforcement strategy and the thermomechanical processing route in enhancing surface strength. The minimum hardness value was observed in the unreinforced PEEK sample compacted at 10 MPa and sintered at 250 °C, aligning with prior studies that indicate the reduced hardness of amorphous or low-crystallinity PEEK due to inadequate molecular chain packing and insufficient structural constraints [16,50]. In contrast, the highest hardness was recorded for the D30–300 composite, produced under 30 MPa compaction pressure and 300 °C sintering temperature. The significant increase in hardness under these processing conditions arises from a combination of densification, enhanced polymer chain reorientation, improved crystalline ordering and strengthened interfacial bonding between the matrix and reinforcement phases [60,61].

The role of boron in the hybrid composite is particularly pronounced. Due to its high intrinsic hardness, chemical inertness and low density, boron acts as an effective hardening phase. In the present system, boron likely hinders localized indentation by restricting polymer chain mobility while simultaneously promoting local crystallization, thereby increasing rigidity [62,63]. Comparable behaviors have been documented in boron carbide (B_4_C) and boron nitride (BN) reinforced polymer composites, where boron-based particles significantly enhance hardness and suppress microplastic deformation [30,44,45].

From the microstructural point of view, the ceramic and metalloid reinforcements dispersed within the PEEK matrix act as load-bearing barriers that impede shear deformation during indentation. These particles prevent polymer chains from undergoing substantial segmental motion, thereby redistributing applied stresses over a larger volume of the composite. When interfacial adhesion between matrix and reinforcement is strong, as achieved here through optimized mixing, compaction and sintering, void formation and interfacial slippage are minimized, contributing further to the elevated hardness. This interfacial integrity is essential, as weak bonding would result in particle pull-out and localized collapse, reducing the composite’s effective hardness [64,65]. From an application standpoint, higher hardness improves not only resistance to abrasion and adhesive wear but also contributes to dimensional stability and enhanced fatigue endurance under cyclic contact conditions. These characteristics are vital in aerospace, automotive and biomedical components, where materials are routinely exposed to sustained surface stresses and long-term mechanical demands [28].

Overall, the experimental results demonstrate that compaction pressure and sintering temperature are decisive factors in determining the hardness of PEEK-based hybrid composites. The synergistic combination of reinforcement addition, especially boron, and thermomechanical densification yields substantial improvements in surface mechanical performance. Future investigations may benefit from exploring the influence of reinforcement morphology and particle size distribution, developing tailored surface treatments to enhance interfacial chemistry, and integrating computational micromechanical models to more deeply elucidate the complex interplay between hardness and tribological behavior in multi-phase PEEK composites.

### 3.5. Tribology

**Figure 9:**
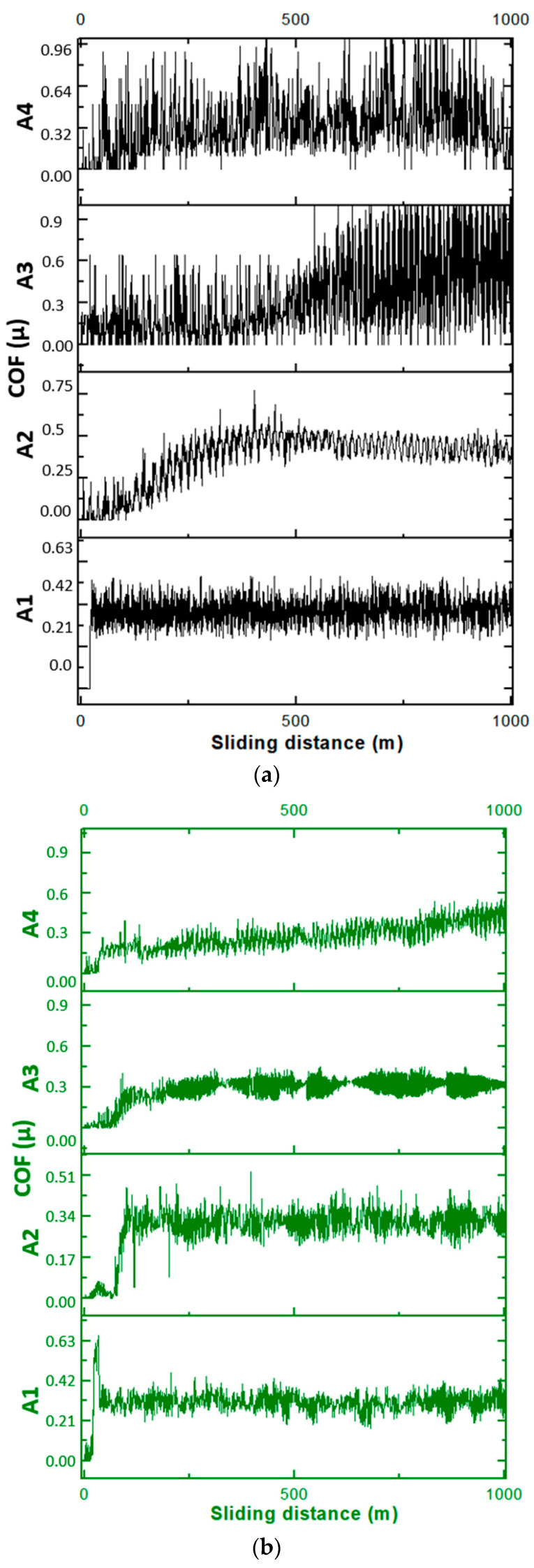

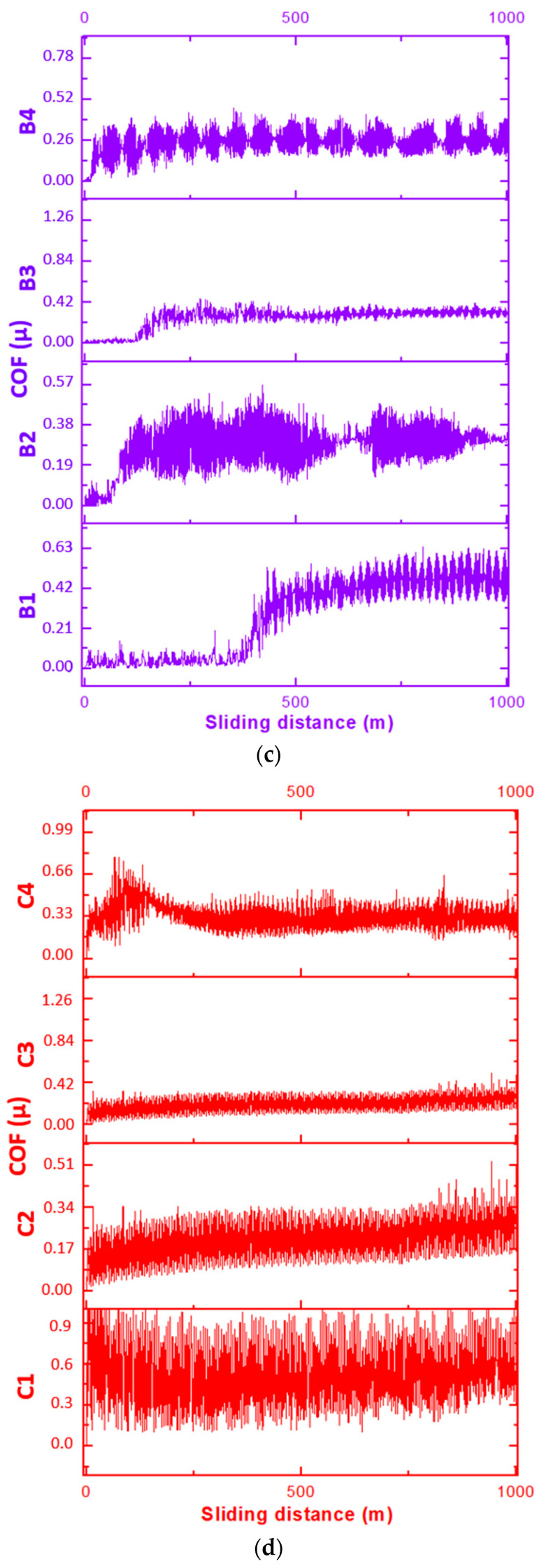

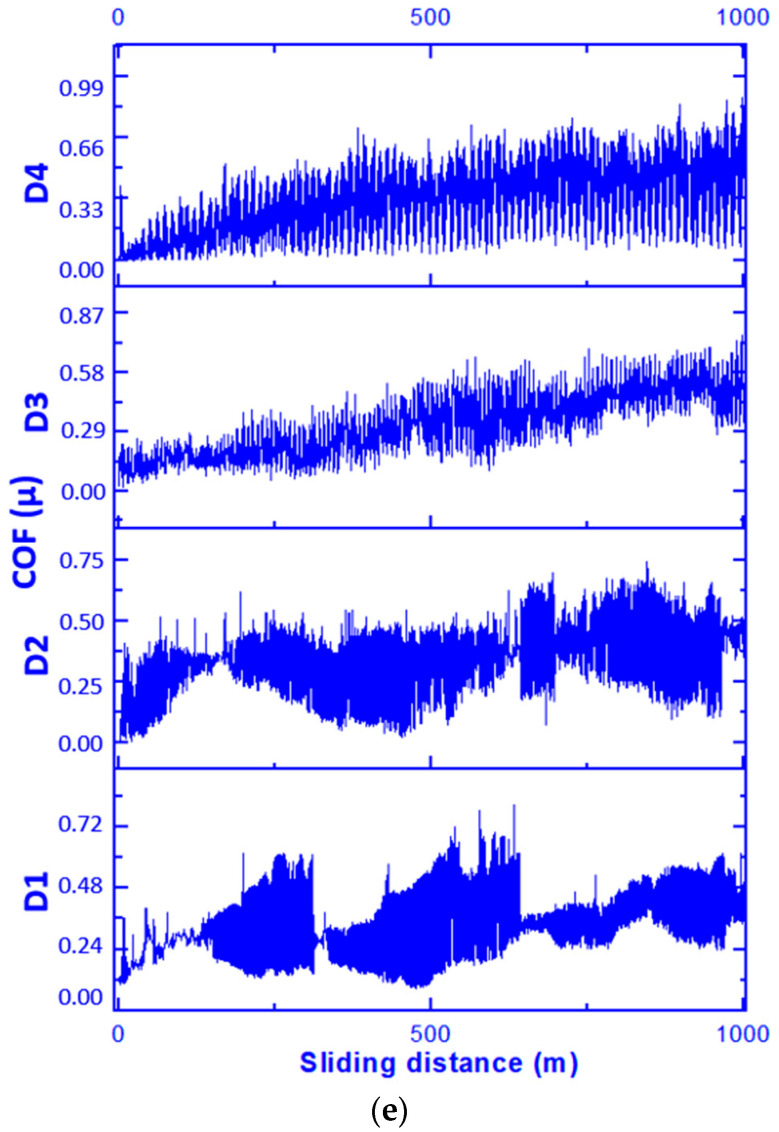
Variation in coefficient of friction with sliding distances for: (**a**) REF, (**b**) A, (**c**) B, (**d**) C, and (**e**) D groups.

**Figure 10:**
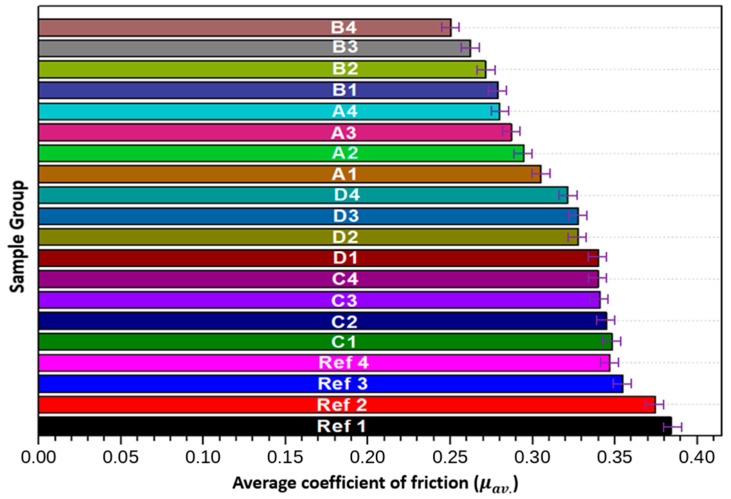
Variation in average coefficient of friction values with differently reinforced groups.

The coefficient of friction (COF) is a critical parameter in determining the tribological performance of materials. It provides insight into the material’s resistance to sliding friction, which directly influences wear resistance and the longevity of composite systems under operational conditions. Figure 9 and Figure 10 present COF data obtained under a 20 N load over a sliding distance The coefficient of friction (COF) is one of the most decisive tribological parameters for evaluating the sliding performance and operational durability of polymer-based composites. It governs energy dissipation at the contact interface and directly influences wear rate, surface damage evolution and long-term reliability under repeated sliding conditions. Figure 9 and Figure 10 present the COF behavior of pure PEEK and hybrid composites under a 20 N normal load and a sliding distance of 1000 m, together with corresponding mass loss data obtained from gravimetric measurements.

As generally established in the literature, surface hardness correlates strongly with tribological response, as harder surfaces typically resist micro-cutting, grooving and abrasive interaction more effectively [48,49]. In this study, the incorporation of graphite (Gr), boron (B), hydroxyapatite (HAp) and zirconium (Zr) yielded a considerable reduction in COF compared with unreinforced PEEK. This improvement indicates that the hybrid reinforcement strategy enhances interfacial stability by strengthening the load-bearing capacity of the surface layer and reducing the severity of asperity interactions.

Graphite, with its lamellar crystalline structure, contributes significantly to friction reduction through its inherent solid lubrication mechanism. The weak van der Waals forces between graphene layers enable facile shearing, thereby forming a lubricating tribofilm that minimizes adhesive interactions and suppresses direct polymer–counterface contact [51]. The presence of boron further enhances this behavior. Previous studies report that boron-containing reinforcements can reduce friction by increasing matrix stiffness, stabilizing the surface under load and contributing to improved thermal management [51,52,53,54,55]. In the present composites, the slight increase in thermal conductivity attributable to Gr and B is particularly beneficial, as it enhances dissipation of frictional heat, limits transient thermal softening of PEEK, and thus supports more stable wear behavior.

The COF curves of the hybrid composites exhibit an initial run-in period followed by stabilization at significantly lower friction levels than pure PEEK. Unreinforced PEEK shows an average COF near 0.38, confirming its tendency toward adhesive friction and its limited capacity to form protective transfer layers, in accordance with earlier findings [53]. By contrast, the hybrid composites, especially those containing Gr and B, transition more rapidly into a steady-state friction regime. This rapid stabilization suggests that the tribofilm formation process is more efficient, enabling the interface to achieve mechanical and chemical equilibrium in a shorter time. Such behavior is advantageous for high-performance engineering applications requiring consistent frictional response over long durations.

**Figure 11:**
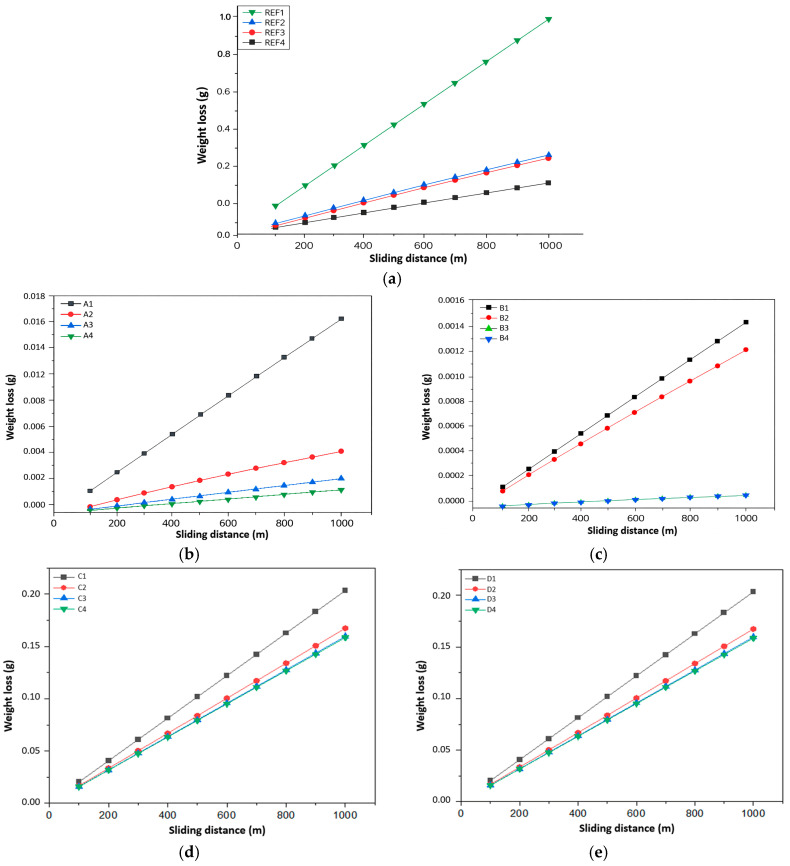
Change in weight loss with sliding distance for the groups: (**a**) REF, (**b**) A, (**c**) B, (**d**) C, and (**e**) D.

Weight loss measurements provided in Figure 11 further validate the enhanced tribological performance of the reinforced composites. All hybrid systems exhibited reduced mass loss relative to pure PEEK, with the most pronounced reductions observed in the Gr- and B-containing specimens. The improved wear resistance arises from the formation of a stable tribofilm composed of graphite debris, boron-containing fragments and compacted polymer material. This composite tribofilm functions as a solid lubricant, reducing surface damage, redistributing interfacial stresses and limiting the extent of material removal. Enhanced heat dissipation in the reinforced systems also plays a role, as it prevents localized softening and degradation of the polymer matrix under sliding-induced temperature rises [58,66].

Although Gr and B significantly improve friction and wear performance, it is important to maintain reinforcement loadings within an optimal range. Excessive filler concentrations can promote particle agglomeration, generating stress concentrations and reducing mechanical integrity [54]. Such effects could counteract the benefits of low friction by promoting premature surface damage or subsurface cracking. Thus, an appropriate balance between reinforcement content, particle size and processing conditions is essential to maximize tribological efficiency without compromising structural stability.

Overall, the tribological results demonstrate that hybrid reinforcement with Gr, B, HAp and Zr significantly enhances COF behavior, wear resistance and thermal stability of PEEK-based composites. The rapid attainment of steady-state friction and the substantial reduction in mass loss position these hybrid materials as strong candidates for applications requiring reliable long-term sliding performance, such as bushings, bearings, implant articulations and high-load mechanical interfaces.

A comprehensive overview of previous research on the tribological behavior of PEEK-based composites is presented in Table 4. The summarized studies highlight the influence of various reinforcements, including carbon fibers, solid lubricants, graphene derivatives, and transition-metal dichalcogenides on friction and wear performance. Across these investigations, a consistent trend emerges: the incorporation of both carbonaceous and solid-lubricating phases into the PEEK matrix results in a significant reduction in friction coefficient and wear rate. This behavior is attributed to enhanced load-bearing capability, improved interfacial stability and the formation of lubricating tribofilms during sliding. The reported findings provide a relevant comparative framework for the tribological responses observed in the present hybrid composite system.

### 3.6. Structure

**Figure 12:**
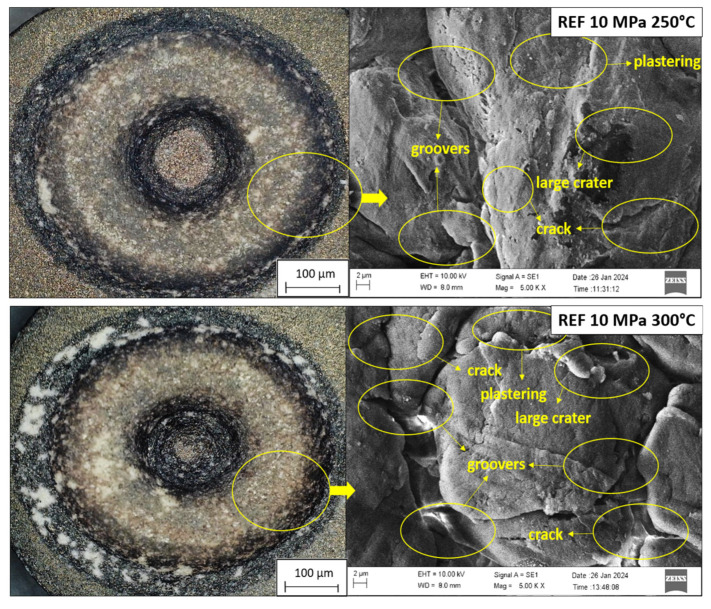

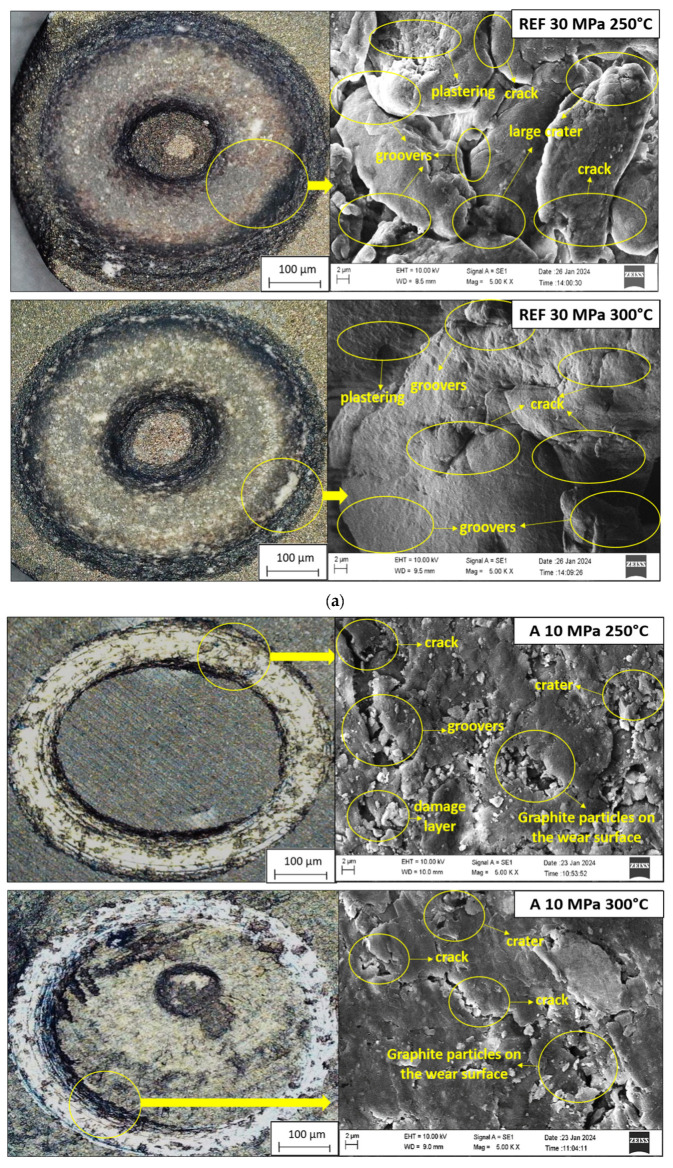

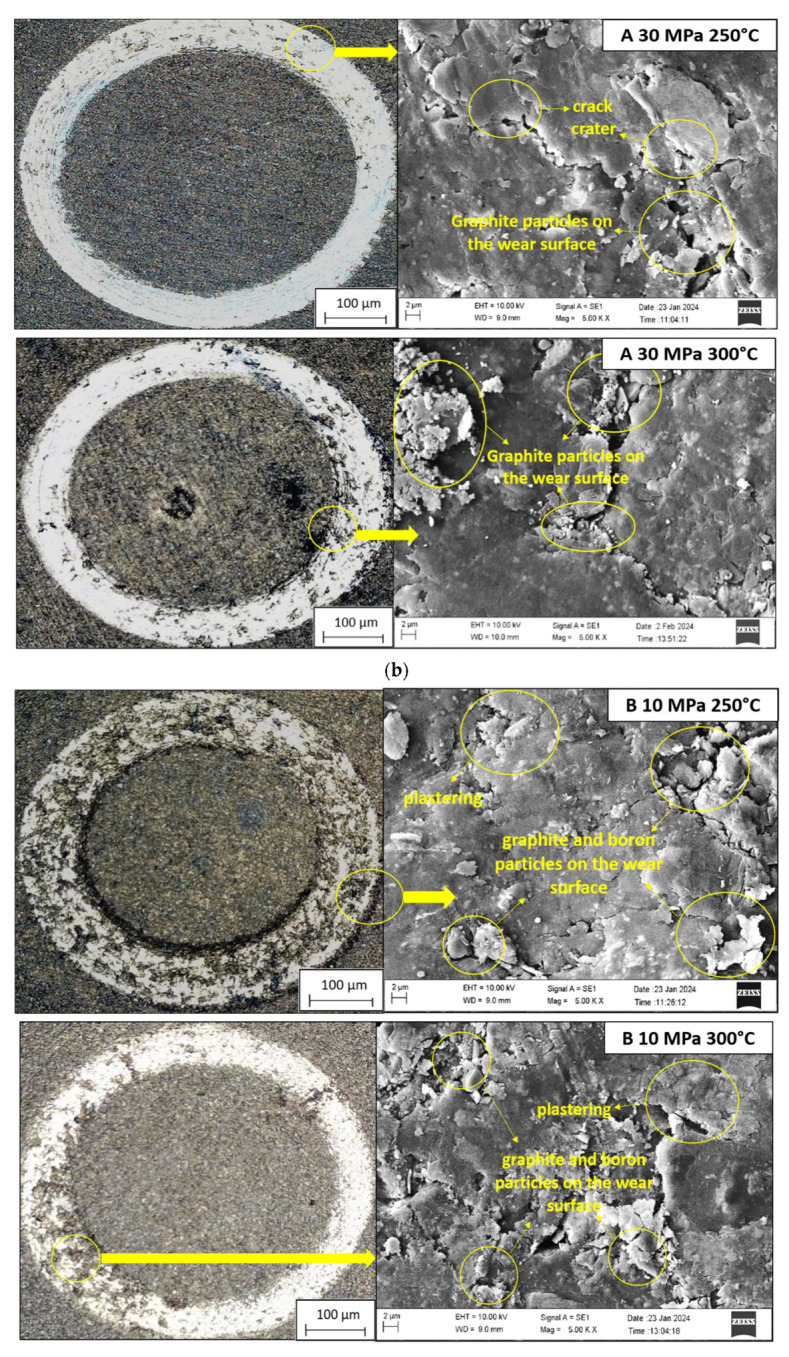

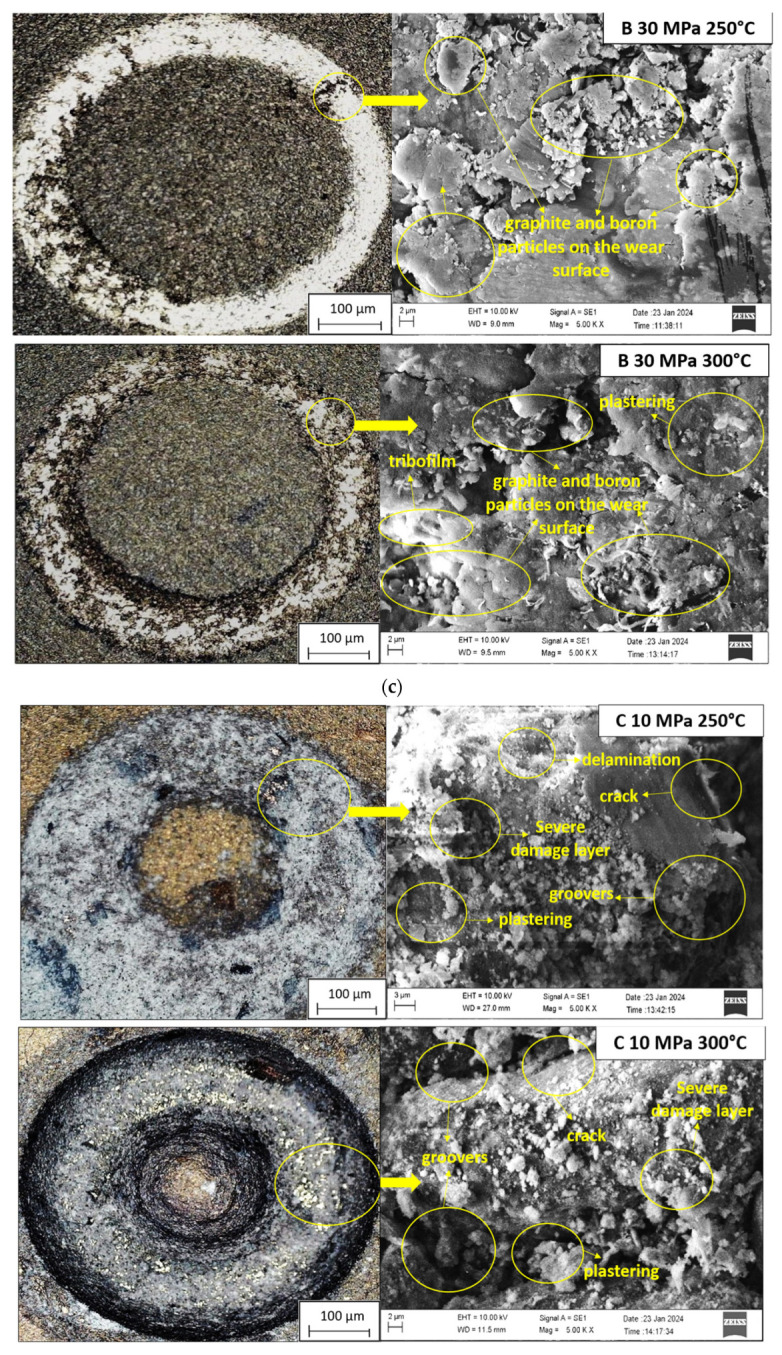

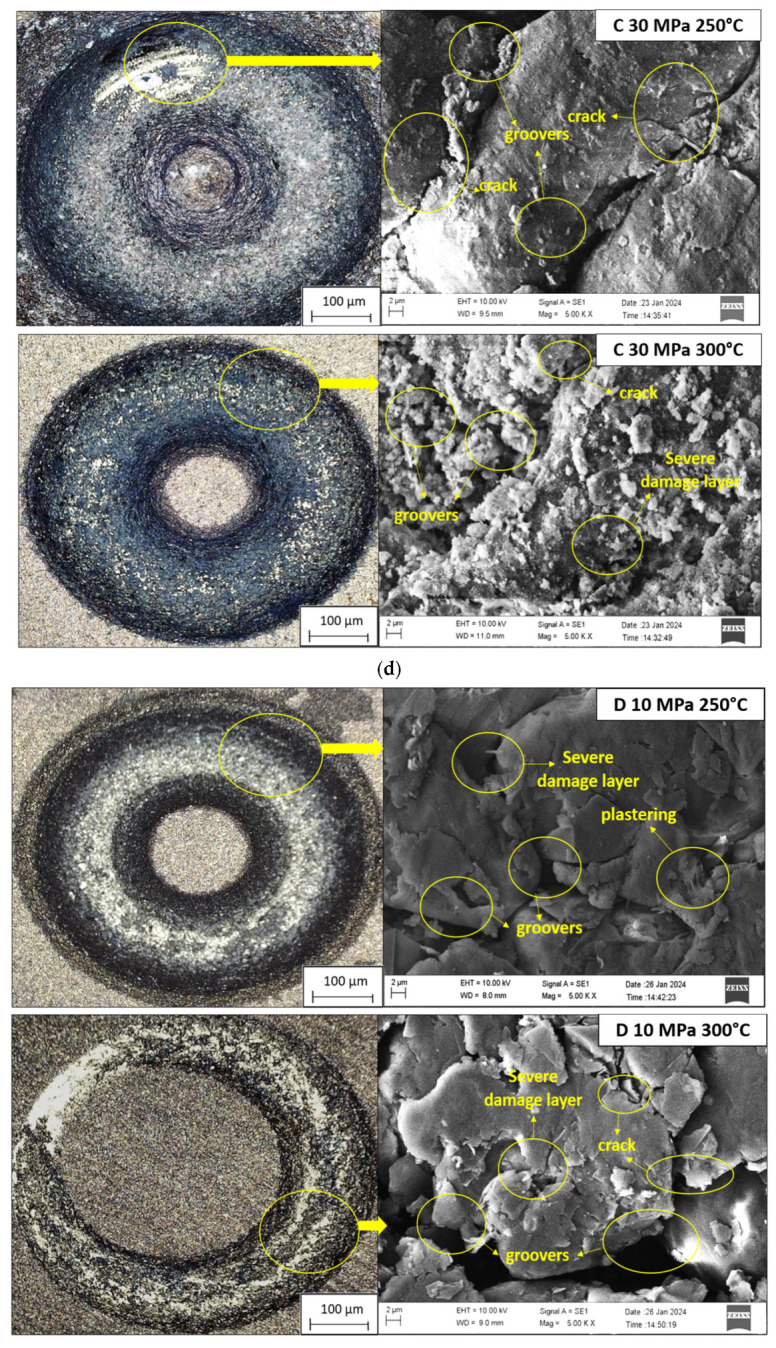

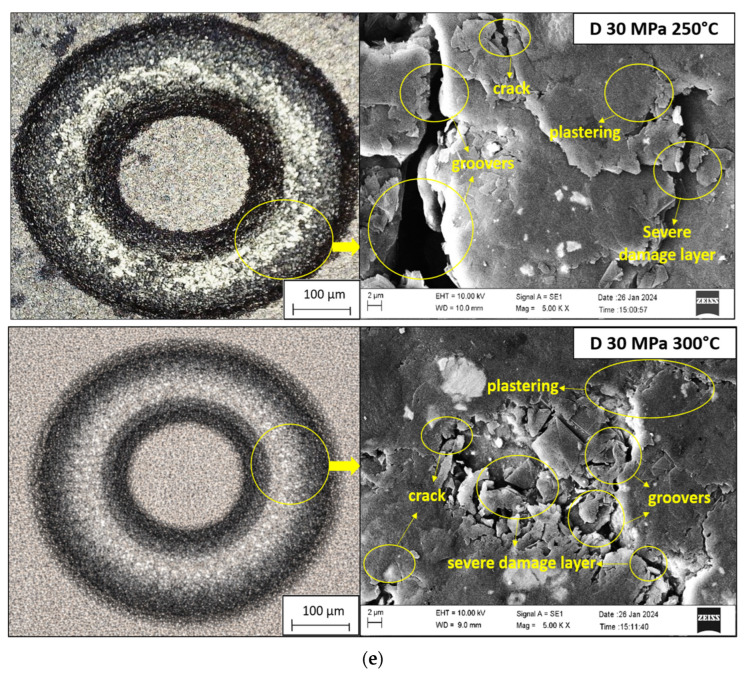
SEM views of the produced PEEK-based hybrid composite groups: (**a**) Ref, (**b**) A, (**c**) B, (**d**) C, and (**e**) D.

Figure 12 presents high-resolution optical and SEM micrographs of the worn surfaces. Together, these results provide a mechanistic link between the measured friction and wear data and the underlying damage evolution at the microstructural level.

For the pure PEEK specimen, the worn surface is characterized by pronounced and continuous grooves aligned with the sliding direction. These grooves arise primarily from the large hardness mismatch between the steel counterface and the relatively soft PEEK matrix. During sliding, asperities on the steel pin penetrate into the polymer surface and generate severe material removal through plowing and micro-cutting mechanisms under the combined action of normal contact stress and tangential frictional shear [40,54]. Detached PEEK fragments accumulate at the interface and form an unstable transfer layer on the steel surface. Although this transfer layer can temporarily reduce friction, its low cohesive strength and poor adhesion render it prone to fragmentation and detachment during prolonged sliding. As a consequence, the contact continuously alternates between phases of partial lubrication and direct metal–polymer interaction, leading to unstable friction behavior and high wear rates [45,46,47,48,49,50].

In stark contrast, the incorporation of Gr and B dramatically modifies the tribological response and the morphology of the worn surfaces. Owing to their high specific surface area and characteristic morphology, both reinforcements contribute to an increase in matrix hardness and compressive strength, effectively reducing the local flow stress. As sliding proceeds and the near-surface polymer is progressively removed, Gr and B particles become exposed at the contact interface and begin to actively participate in load-bearing. Their presence stabilizes the contact by shielding the PEEK matrix from direct asperity penetration and promoting the in situ development of a more robust tribolayer. This tribolayer acts as a physical and mechanical barrier that limits metal–polymer adhesion and mitigates severe micro-cutting [47,48].

The observed transition in wear mechanisms from a mixed adhesive/abrasive regime in pure PEEK to a predominantly fatigue and tribofilm-controlled regime in Gr- and B-reinforced composites is consistent with these morphological changes. In the reinforced systems, strong matrix–filler interfacial bonding, as established in the microstructural analyses, reduces the likelihood of particle pull-out and ensures that reinforcements remain anchored during sliding. This enhances the structural integrity of the load-bearing surface layer and delays the formation of large-scale delamination cracks [50,51].

In pure PEEK and in composites with suboptimal interfacial bonding, the worn surfaces exhibit extensive plastic flow, severe adhesive wear residues, and deep furrows, often accompanied by fatigue-induced delamination and exfoliation of material fragments. Entrapped hard debris between the sliding partners further intensifies abrasive wear, generating secondary scratches and micro-grooves. By contrast, the Gr–B hybrid composites display considerably smoother surfaces with fewer and shallower grooves, reduced micro-cracking and more localized damage. In particular, worn surfaces of groups A and B show only occasional spalling pits, relatively shallow parallel grooves and “water-wave-like” scratch patterns, indicating a more benign wear regime dominated by mild abrasion and stabilized tribofilm formation rather than catastrophic surface failure [55,56,57,58].

The beneficial effect of boron is especially noteworthy. In Gr–B hybrid composites, layered boron-containing structures with weak interlayer bonding enable the development of a continuous and stable lubricating film at the sliding interface. This film progressively fills surface asperities and wear scars, redistributing contact stresses and embedding loose wear debris. As a result, the surface becomes smoother and more conformal, further enhancing abrasion resistance and reducing the tendency for crack nucleation. The self-lubricating and film-forming character of boron-based phases is in line with previous findings, which report the formation of robust tribolayers via weak interlayer bonding and interfacial shear [70,71].

Composites reinforced with Zr and HAp also exhibit improved wear resistance compared to pure PEEK, but their performance remains inferior to Gr- and B-containing systems. SEM observations reveal that Zr- and HAp-reinforced surfaces still show evidence of micro-cracking, localized spallation and relatively more pronounced grooves than Gr–B hybrids. This behavior can be attributed to less effective interfacial bonding between PEEK and Zr/HAp during pressing and sintering. Weak interfaces promote partial pull-out of ceramic particles and the generation of hard wear debris, which then act as third bodies and contribute to abrasive damage. Such phenomena are consistent with previous reports indicating that insufficient bonding during sintering leads to reinforcement detachment and increased surface degradation [72].

The inherent limitations of unmodified PEEK also play a role in these observations. Due to its relatively low thermal conductivity, frictional heating generated at the sliding interface is not efficiently dissipated, leading to localized temperature rises, thermal softening of the matrix and an associated increase in COF and wear rate [14,40,54]. The studies by Sariyev et al. [8] have highlighted that thermal instability is a key factor controlling the wear performance of PEEK-based materials. In the present work, the enhancement of thermal conductivity and surface stiffness through Gr and B incorporation mitigates localized thermal softening and supports a more stable tribological response, in agreement with observations by Sathishkumar et al. [73] for graphene-reinforced systems.

Literature findings on the continuous lubricating function of boron are in excellent agreement with the present results. Gül et al. [70] showed that boron-based fillers can develop a persistent interfacial tribolayer through weak interlayer bonding, significantly improving wear performance.

Overall, among all tested formulations, the PEEK/Gr–B hybrid composites exhibit the lowest COF and the most favorable wear surface morphologies, indicating a highly effective synergy between the lamellar sliding mechanism of graphite and the self-lubricating, tribofilm-forming capability of boron. This dual-phase reinforcement strategy stabilizes the interfacial tribolayer, homogenizes the stress distribution and suppresses stress concentration zones, in line with the observations of Şenel et al. [74]. As a result, the Gr- and B-reinforced PEEK composites demonstrate substantially reduced wear rates and friction coefficients relative to pure PEEK.

In summary, the post-wear microstructural examinations corroborate the macroscopic tribological data and clearly show that the improved performance of the hybrid composites is governed by the formation of continuous, in situ generated lubricating films; enhanced load-bearing capacity due to exposed reinforcements; and strengthened matrix–filler interfaces that suppress severe adhesive and abrasive wear. The SEM images in Figure 12 visually confirm these conclusions by revealing less surface damage, shallower wear tracks and more uniform surface topographies in the hybrid-reinforced samples, particularly in the PEEK/Gr–B systems.

### 3.7. Compression Tests

Compression testing was carried out to assess the mechanical integrity, stiffness, and load-bearing capability of the hybrid PEEK-based composites under uniaxial compressive loading. Such characterization is crucial for polymer matrix composites, particularly those intended for structural or biomedical applications where components must withstand sustained compressive forces without excessive deformation or premature failure. The elastic modulus, a key descriptor of the material’s resistance to elastic deformation, was extracted from the linear region of the stress–strain curves, providing insight into the stiffening effect imparted by the various reinforcements.

**Figure 13:**
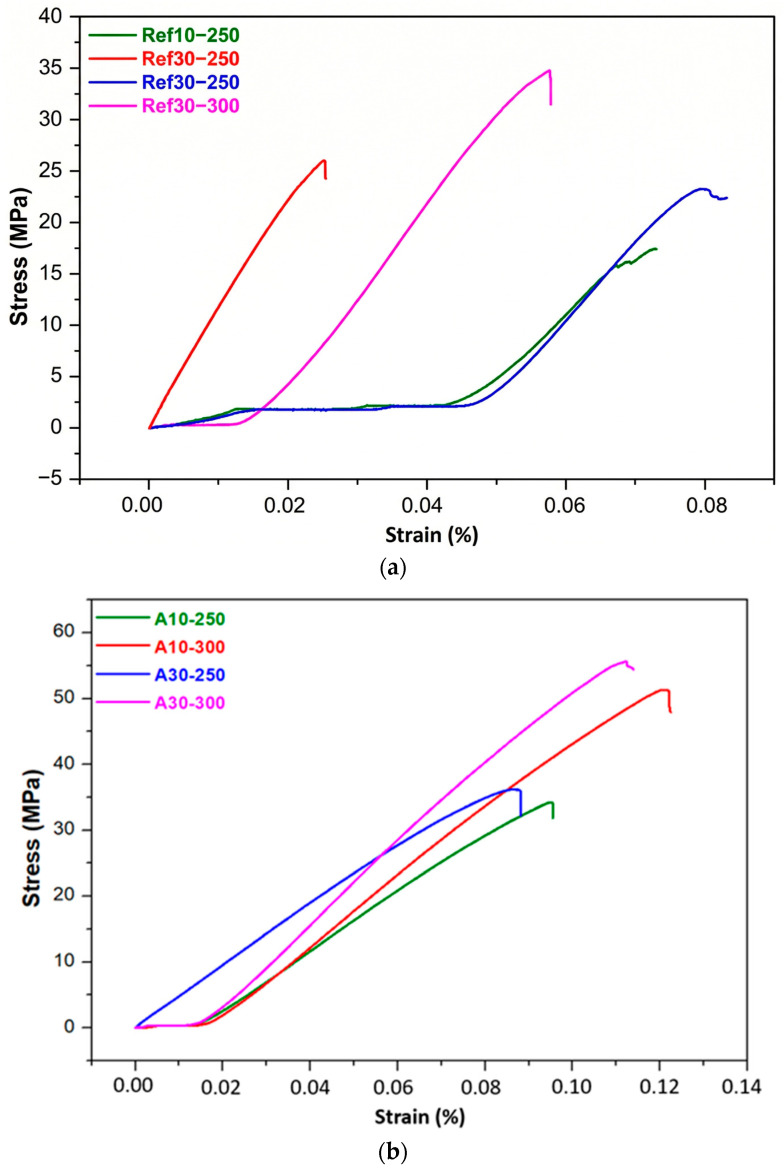

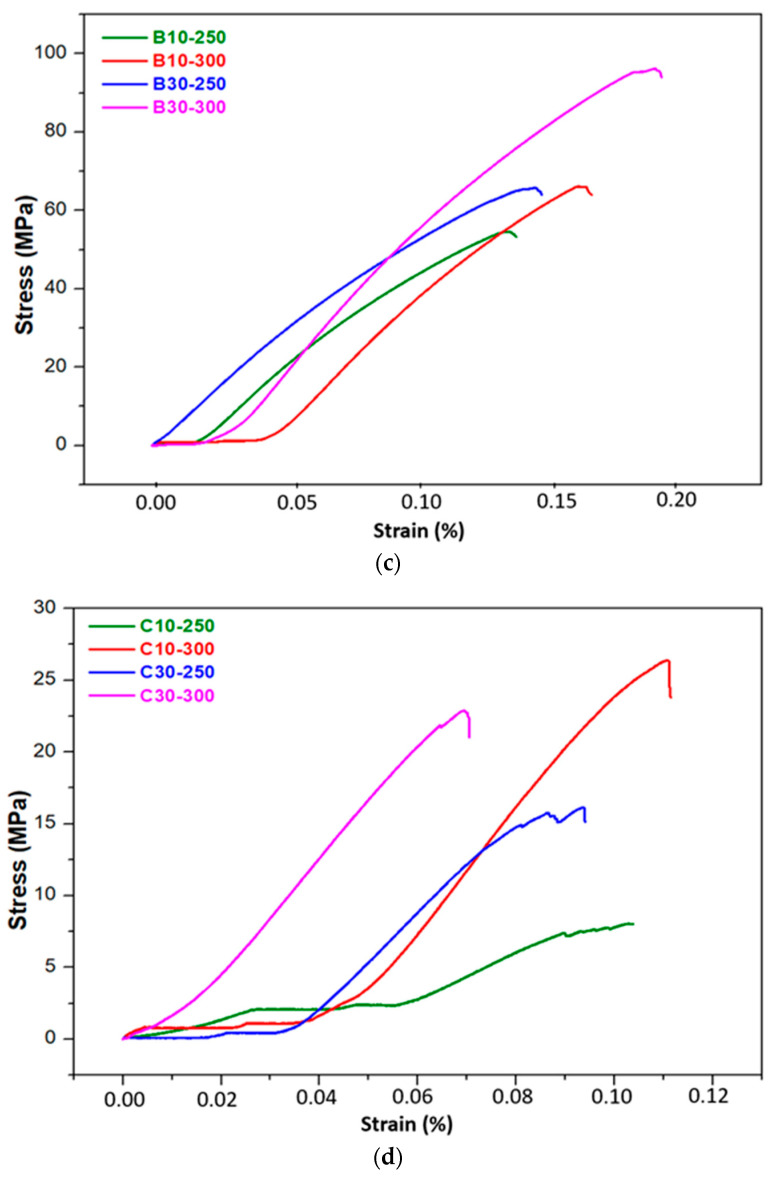

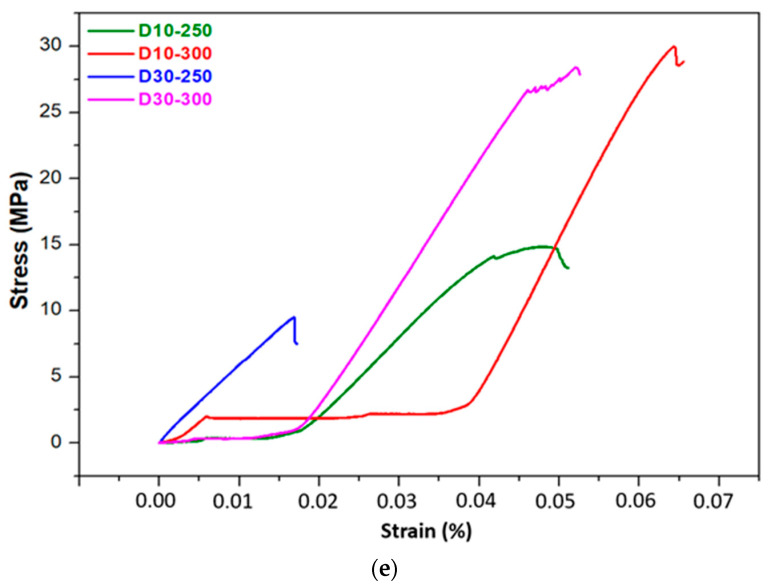
Stress–strain diagrams of the produced hybrid composites: (**a**) REF, (**b**) A, (**c**) B, (**d**) C and (**e**) D groups.

The tests were conducted at room temperature (22 ± 2 °C), and at a constant crosshead speed of 0.5 mm/min was employed to ensure uniform strain rate conditions. Cylindrical specimens with dimensions Ø10 × 15 mm were tested, and at least three replicates were examined for each composite group to ensure statistical significance. The corresponding load–displacement and stress–strain responses, illustrated in Figure 13, reveal clear distinctions in elastic response, yield behavior and failure modes among the different formulations.

Unreinforced PEEK exhibited a maximum compressive strength of approximately 35 MPa, which aligns with the expected mechanical behavior of semi-crystalline thermoplastics whose load-bearing capacity is limited by chain mobility and moderate crystallinity. By contrast, all reinforced specimens demonstrated substantial improvements in compressive performance, with the B4 composite containing 80 wt.% PEEK, 10 wt.% graphite and 10 wt.% boron achieving a peak compressive strength of 96 MPa, nearly a threefold increase relative to neat PEEK. This enhancement stems from the high intrinsic stiffness of graphite and boron, their ability to transfer load across the matrix–filler interface efficiently, and the microstructural densification promoted by well-dispersed particulate phases. Comparable trends have been reported by Bera et al. [75], who observed that carbon-based fillers improve compressive strength through their high modulus and strong interfacial interactions with polymer chains. Boron’s contribution to thermal stability and creep resistance, previously reported by Uzay [76], further supports the improved resistance to compressive deformation observed here.

All reinforced samples displayed a largely linear stress–strain response prior to failure, indicating a shift toward more brittle behavior at increased filler concentrations. The rise in compression modulus across the reinforced systems reflects the stiffening effect produced by the ceramic and carbonaceous fillers. However, the performance is highly dependent on dispersion quality. Uniformly distributed particles facilitate effective load transfer, delay crack initiation and prevent the formation of localized stress concentrations. Conversely, particle agglomeration can act as a mechanical flaw that precipitates premature failure, a phenomenon consistent with the findings of Rani et al. [77], which emphasize the central role of dispersion in determining the reliability of thermoplastic composites.

The filler type significantly influenced the compressive properties. Graphite–boron hybrid systems outperformed HAp-reinforced composites, owing to the superior hardness, stiffness and interfacial bonding of Gr and B. In contrast, HAp additions tended to form localized clusters, generating microstructural heterogeneity and increasing the likelihood of stress concentration and early crack formation. Similar clustering-induced weakening has been noted by Cheang et al. [78], who reported diminished load-bearing capacity when HAp is not adequately dispersed. Such limitations could be alleviated through surface modification, such as silane coupling, aimed at improving HAp–PEEK interfacial compatibility.

Zirconium-reinforced composites exhibited moderate improvements in compressive strength but remained inferior to the Gr–B systems. This more limited enhancement may be attributed to the intrinsic stiffness of Zr particles, which promotes localized constraint while reducing matrix ductility, coupled with potential mismatches in interfacial chemistry. As highlighted by Rucki et al. [79], Zr-based fillers can significantly strengthen polymer matrices only when sintering temperature, pressure and interfacial conditions are precisely optimized. Tailored surface treatments and modified sintering schedules may therefore be required to unlock the full reinforcing potential of zirconium in PEEK systems.

Taken together, these results demonstrate that graphite and boron provide the most effective reinforcement strategy for improving both compressive strength and modulus in PEEK-based composites. Their synergistic interaction, combined with favorable dispersion and strong interfacial bonding, results in composites with markedly enhanced mechanical durability suitable for structural and tribological applications. To further advance these systems, future work should focus on refining filler dispersion, tailoring interfacial chemistry and conducting multi-scale microstructural analyses to more deeply understand load transfer and failure mechanisms under compressive stress.

The experimental results clearly show that the hybrid composites compacted at 30 MPa and sintered at 300 °C exhibit the highest compressive strength among all tested configurations. The superior mechanical response can be attributed to the combined effects of enhanced particle packing density, improved interfacial adhesion and increased matrix densification that arise under elevated compaction pressures and sintering temperatures. By promoting closer contact between PEEK and the reinforcement phases, these processing conditions reduce residual porosity and facilitate more efficient load transfer across the matrix–filler interface. These observations are consistent with previous studies reporting that higher compaction pressures and sintering temperatures significantly strengthen polymer-based composites by increasing crystallinity, refining microstructure and promoting stronger inter-phase bonding [80,81]. Zhou et al. similarly found that sintering PEEK composites above 280 °C enhances crystalline ordering and interfacial compatibility, contributing to higher compressive strength [82]. Kim et al. further demonstrated that elevated sintering temperatures drive microstructural densification, resulting in improved strength and wear resistance in ceramic-reinforced PEEK systems [82,83].

**Figure 14:**
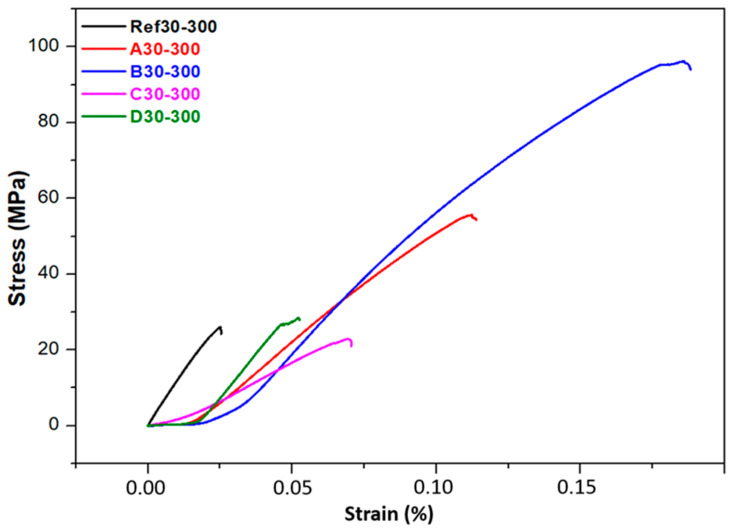
Stress–strain diagrams of the produced hybrid composites.

Fractographic analysis after compression (Figure 14) revealed the formation of crimp bands along two distinct shear planes, indicating that microbuckling and fiber crimping are the dominant failure modes in these hybrid composites. This failure pattern is characteristic of systems where stiff reinforcements constrain the polymer matrix, leading to stress localization and shear-band initiation under axial loading. These fracture features align with the findings of Ma et al., who emphasized the importance of filler–matrix interfacial quality in governing deformation mechanisms in hybrid composites [84]. The observed shear-band-induced fracture surfaces closely resemble those reported for high-strength particle- and fiber-reinforced polymers, where constrained plastic deformation intensifies local stresses and accelerates shear propagation during catastrophic failure [85,86].

Interestingly, the compressive strength values achieved by the Gr–B-reinforced composites fall within the range of human cortical bone (106–215 MPa), highlighting their strong potential for biomedical applications. This alignment suggests that such composites could serve as promising candidates for load-bearing implants, spinal fusion cages and bioactive scaffolds. Prior studies by Li et al. demonstrated that PEEK reinforced with bioceramics such as Hap and ZrO_2_ can achieve compressive strengths comparable to natural bone, supporting their suitability for osteoconductive medical devices [83]. Ma et al. likewise reported that PEEK’s inherent biocompatibility, radiolucency and chemical stability make it an attractive alternative to metallic implant materials when combined with appropriate reinforcements [84]. In the present study, the enhancements observed in Gr–B composites arise from three synergistic mechanisms: the high load-bearing capacity of the reinforcements, the efficient transfer of compressive stress due to improved filler–matrix adhesion and the formation of a self-lubricating interfacial layer that suppresses crack initiation and delays failure under repeated loading [85,86,87].

Comparative analysis with literature further supports these conclusions. Zhang et al. demonstrated that adding 10 wt.% SiO_2_ nanoparticles improved compressive strength by 25.3% due to enhanced dispersion and stronger interfacial bonding; however, higher loadings (20–30 wt.%) caused significant reductions in strength by 34.1% and 124.1%, respectively, because of agglomeration-induced stress concentrations [87]. These findings align with the results of Singh et al. (2021), who showed that excessive nanofiller content compromises mechanical integrity by increasing brittleness and promoting premature failure [85].

The addition of ZrO_2_ resulted in moderate improvements in compressive strength, though the effect was markedly weaker than that observed in the Gr–B composites. This behavior is attributed to the brittle nature of zirconia, which reduces overall ductility and increases the likelihood of microcrack formation at the filler–matrix interface. Such cracks disrupt stress transfer and limit the overall contribution of ZrO_2_ to load-bearing efficiency [84]. Hap-reinforced composites also displayed comparatively lower compressive strength. This reduction stems from particle agglomeration and insufficient dispersion, which foster localized stress concentrations and facilitate premature crack initiation under compressive loads. These outcomes are consistent with Pan et al. (2015), who reported that excessive or poorly dispersed HAp in PEEK matrices accelerates fracture through interfacial instability [86].

**Figure 15:**
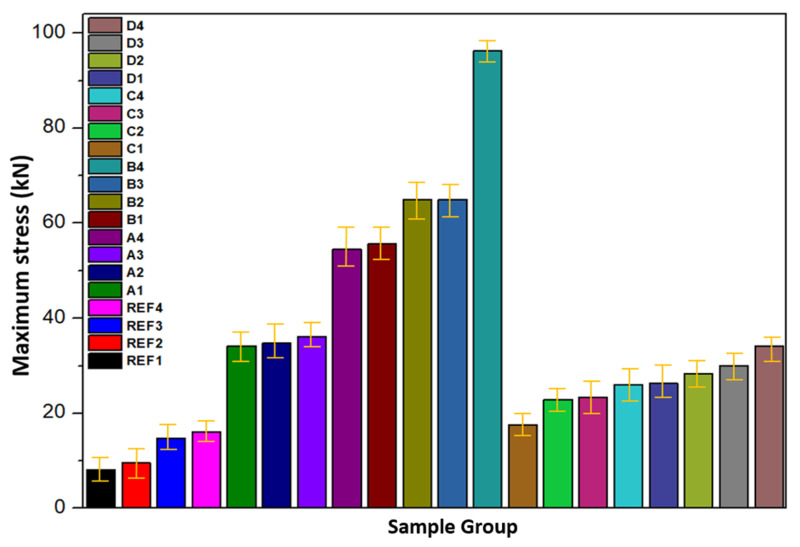
Maximum stress values of the reinforced composite groups.

Taken together, these results confirm that graphene and boron provide the most effective strengthening mechanism among all examined reinforcements. Their superior interfacial compatibility, high intrinsic stiffness and self-lubricating properties synergistically enhance compressive strength while maintaining acceptable levels of ductility. Improved filler–matrix adhesion mitigates crack propagation, while the reduced porosity and refined microstructural uniformity achieved under optimized compaction and sintering conditions contribute further to mechanical reliability. The maximum compressive strength values and corresponding fracture morphologies, shown in Figure 14 and Figure 15, underscore the strong potential of Gr–B-reinforced PEEK hybrids for next-generation structural and biomedical components requiring high compressive strength, dimensional stability and long-term durability.

## 4. Conclusions

This study has systematically elucidated the effects of Gr, B, HAp and Zr reinforcements on the microstructural, mechanical and tribological behavior of PEEK-based hybrid composites produced via PM. XRD and FT-IR analyses confirmed that all reinforcement phases were successfully incorporated into the PEEK matrix without detectable phase decomposition or undesirable reaction products, indicating that the selected compaction and sintering parameters preserved the chemical integrity of both the polymer and the fillers.

SEM and EDX investigations demonstrated a fairly uniform dispersion of the reinforcing particles within the PEEK matrix, with greater compaction pressure (30 MPa) and sintering temperature (300 °C), resulting in enhanced interfacial bonding, reduced agglomeration, and decreased porosity. Among all formulations, the Gr–B hybrid composite exhibited the most pronounced strengthening response, with a hardness increase of approximately 240% relative to neat PEEK. This substantial enhancement is attributed to the high intrinsic hardness and stiffness of Gr and B, their favorable interfacial compatibility with the matrix and their ability to restrict localized plastic deformation, thereby enabling more efficient stress transfer throughout the composite.

From a tribological standpoint, the B-containing hybrid composite exhibited a 34.7% reduction in the coefficient of friction and an approximately 90% decrease in wear-induced mass loss compared to unreinforced PEEK. These improvements are associated with the in situ formation of a stable, self-lubricating tribolayer at the sliding interface, which limits direct asperity contact, reduces adhesive and abrasive wear contributions and promotes a shift toward a more fatigue-controlled wear regime under dry sliding conditions.

The combination of Gr and B was found to be extremely effective at stabilizing the contact interface, improving surface integrity, and suppressing frictional fluctuations. In compression, the Gr-B-reinforced composite had the highest compressive strength at 96 MPa. In contrast, HAp-containing composites exhibited comparatively lower mechanical performance, largely due to particle clustering and incomplete dispersion, which promoted localized stress concentrations and premature failure. Zr-reinforced materials showed moderate strength gains but a reduction in ductility, highlighting a trade-off between stiffness and toughness within these hybrid systems.

Collectively, the findings demonstrate that hybrid reinforcement strategies based on Gr and B are particularly effective in simultaneously enhancing hardness, wear resistance and compressive strength in PEEK-based composites, while maintaining structural integrity. The Gr-B-reinforced PEEK system hybrid composites emerge as a promising candidate for high-demand applications that require high compressive load capacity, low friction, and long-term dimensional stability, such as spinal cages, orthopedic fixation components, and tribological elements in aerospace and automotive assemblies. Future work should concentrate on further optimizing filler dispersion and volume fraction, tailoring the Gr/B/HAp/Zr ratios for application-specific property profiles and integrating advanced processing routes such as additive manufacturing, surface functionalization or graded architectures to refine interfacial characteristics and enable multifunctional performance, particularly in the context of next-generation structural and biomedical PEEK-based devices.

## Figures and Tables

**Table 1:** The produced composite groups’ percentage contribution rates.

Groups	PEEK (wt.%)	Gr (wt.%)	B (wt.%)	HAp (wt.%)	Zr (wt.%)
Ref	100	-	-	-	-
A	90	10	-	-	-
B	80	10	10	-	-
C	80	10	-	10	-
D	80	10	-	-	10

**Table 2:** Powder purity and particle sizes used during composite synthesizing.

Material	PEEK	Gr	B	Zr	HAp
Purity (%)	99.9	99.9	99.9	99.9	99.9
Particle size (µm)	0 < PEEK < 50	0 < Gr < 35	0 < B < 35	0 < Zr < 35	0 < HAp < 35
Brand	Sigma-Aldrich, (Darmstadt, Germany)	Nanografi, (Ankara, Turkey)	Nanografi, (Ankara, Turkey)	Nanografi, (Ankara, Turkey)	Nanografi, (Ankara, Turkey)

**Table 3:** Cold press and sintering process parameters of the produced PEEK-sample groups.

Sample Nomenclature		Sample Composition	Sintering Temperature (°C)	Cold Press Pressure (MPa)
Ref 1	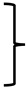	PEEK (100%)	250	10
Ref 2			300	10
Ref 3			250	30
Ref 4			300	30
A1	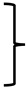	(90%PEEK + 10%Gr)	250	10
A2			300	10
A3			250	30
A4			300	30
B1	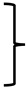	(80%PEEK + 10%Gr + 10%B)	250	10
B2			300	10
B3			250	30
B4			300	30
C1	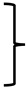	(80%PEEK + 10%Gr + 10%HAp)	250	10
C2			300	10
C3			250	30
C4			300	30
D1	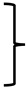	(80%PEEK + 10%Gr + 10%Zr)	250	10
D2			300	10
D3			250	30
D4			300	30

**Table 4:** Literature reviews on the tribological properties of composites featuring PEEK matrix.

Composite Composition	Results Obtained	Literature
**PEEK–CF**	Carbon fibers act as primary load-bearing elements, thereby reducing the polymer matrix contribution to friction in CF-reinforced PEEK systems.	[67]
**PEEK–MoS_2_–WS_2_**	Increasing concentrations of solid lubricants lowered friction coefficients; even small additions (0.5–1 wt.%) of micro/nano MoS_2_ and nano WS_2_ enhanced wear resistance.	[50]
**PEEK**	Under a 20 N load, the pure PEEK sample exhibited a COF of ~0.40.	[68]
**PEEK–GNP**	Wear tests showed a 38% reduction in COF and an 83% reduction in wear factor due to graphene nanoplatelet reinforcement.	[16]
**PEEK/GO and PEEK/MoS_2_**	The addition of GO reduced COF by 18%, while MoS_2_ provided a 15% reduction, demonstrating the effectiveness of both fillers in enhancing lubrication.	[69]

## Data Availability

Data will be made available on request.
